# Programming Photodegradability into Vinylic Polymers via Radical Ring‐Opening Polymerization

**DOI:** 10.1002/anie.202213511

**Published:** 2023-01-09

**Authors:** Phuong T. Do, Berwyck L. J. Poad, Hendrik Frisch

**Affiliations:** ^1^ School of Chemistry and Physics Queensland University of Technology 2 George Street Brisbane QLD-4000 Australia; ^2^ Centre for Materials Science Queensland University of Technology 2 George Street Brisbane QLD-4000 Australia; ^3^ Central Analytical Research Facility Queensland University of Technology 2 George Street Brisbane QLD-4000 Australia

**Keywords:** Coumarin, Ion Mobility-Mass Spectrometry, Photodegradable Polymer, Ring-Opening Polymerization, Sunlight Degradable Polymer

## Abstract

Incorporation of photolabile moieties into the polymer backbone holds promise to remotely‐control polymer degradation. However, suitable synthetic avenues are limited, especially for radical polymerizations. Here we report a strategy to program photodegradability into vinylic polymers by exploiting the wavelength selectivity of photocycloadditions for radical ring‐opening polymerization (rROP). Irradiation of coumarin terminated allylic sulfides with UVA light initiated intramolecular [2+2] photocycloaddition producing cyclic macromonomers. Subsequent RAFT‐mediated rROP with methyl acrylate yielded copolymers that inherited the photoreactivity of the cyclic parent monomer. Irradiation with UVB initiated efficient photocycloreversion of the coumarin dimers, causing polymer degradation within minutes under UVB light or days under sunlight exposure. Our synthetic strategy may pave the way to insert photolabile linkages into vinylic polymers, tuning degradation for specific wavelengths.

## Graphical abstract

Cyclic allylic sulfides were obtained through intramolecular photocycloaddition of coumarin under UVA light. Subsequent radical ring‐opening copolymerization with MA using RAFT, incorporated the coumarin dimers into the backbone of polymers. By decreasing the irradiation wavelength to UVB light or exposing the polymer to sun light, the photocycloreversion of the imbedded coumarin dimers initiated photodegradation of the polymer.

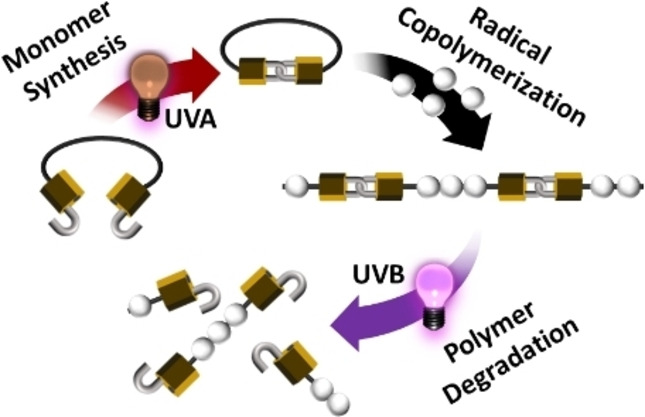

Synthetic polymers have become indispensable to human life as they are used for a wide variety of applications, ranging from daily use such as shopping bags to high end applications such as pharmaceutical use. Stimuli‐responsive polymers, where the physical and chemical properties change under the influence of external stimuli (e.g. pH, temperature, electric charge, enzymatic activity or light exposure), allow for design of soft‐matter materials with remote‐controlled properties.^[1]^ Light is a particularly advantageous stimulus, allowing for exceptional spatiotemporal control under non‐invasive conditions.^[2]^ Consequently, light‐degradable polymers find a wide range of applications, including environmentally benign plastics,^[3]^ controlled release systems^[4]^ or photo patterning.^[5]^ While ultraviolet (UV) light is well known to cause weathering degradation of polymer materials, pioneering mechanistic studies on UV initiated degradation focused on ketone polymers.^[6]^ To enable on‐demand polymer photodegradation, the presence of photolabile groups within the polymer backbone is key. Incorporation of photolabile groups can be readily achieved via polycondensations, giving rise to photodegradable polyurethanes,^[7]^ polycarbonates,^[8]^ polyureas and poly ethers.^[9]^ Photolabile functional groups can also be inserted into polymer backbones through [2+2] photocycloddition based photopolymerization of bivalent building blocks, which can be reverted at shorter wavelengths.[^10^, ^11^] While highlighting the wide application potential of light degradable polymers, incorporation of photolabile linkers into polymer main chains is generally limited to step‐growth polymerizations.

Chain‐growth polymerizations of radical polymerizations are arguably the most widely applied polymerization techniques in both fundamental research and commercial polymer production (representing 45 % of global polymer production).^[12]^ However, radical polymerization of vinylic monomer feedstock exclusively yields polymers with all carbon backbones, preventing direct incorporation of photodegradable moieties. Radical ring‐opening polymerization (rROP) has been recognized as a powerful and versatile tool in polymer synthesis, allowing for the incorporation of functional groups into vinylic polymer chains.^[13]^ Recent advances in rROP have allowed for the ring‐opening to occur independently on the strain of the ring, with finer control over the polymer structure and polymerization that broadens the application of rROP to many functional groups.^[14]^

Herein, a synthetic strategy to program inherent photodegradability into vinylic polymer main chains is reported as the first example of deploying rROP for photo responsive polymer synthesis (Scheme 1). A linear allylic sulfide **C2** with two coumarin end groups was cyclized via intramolecular [2+2] photocycloaddition to afford a cyclic monomer **C3**. Prior work of Hawker and Evans^[14a]^ has shown that cyclic allylic sulfides enable radical ring‐opening polymerization without relying on the strain of the cyclic ring. Incorporation of a phenyl group adjacent to the C−S bond provides electronic deactivation of the newly formed double bond in in the resulting polymer that limits its reactivity, increasing the control over the polymerization as shown by Niu et al.^[14b]^ Radical copolymerization of **C3** with a vinylic monomer yielded photoreactive cyclobutane moieties within the resulting polymer backbone. Upon the UVB exposure, cyclobutane ring cleavage of the imbedded coumarin dimers led to polymer chain scission.

**Scheme 1:**
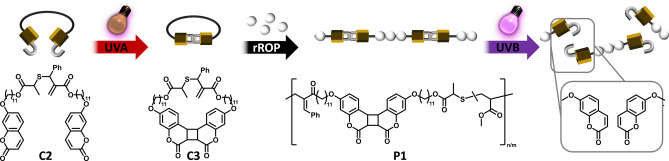
Schematic representation of photodegradable polymer synthesis and its photodegradation: UVA irradiation of allylic sulfide **C2** initiates intramolecular [2+2] photocycloaddition of terminal coumarin end groups yielding cyclic allylic sulfide **C3**; the radical ring‐opening copolymerization between **C3** and a vinylic monomer results in the incorporation of coumarin dimers into the main chain of the resulting polymer **P1**; UVB light exposure initiates the cycloreversion of the imbedded coumarin dimer causing polymer degradation.

To obtain the cyclic monomer **C3**, solutions of linear allylic sulfide **C2** (2 mg mL^−1^) in acetonitrile were irradiated with broadband UVA light to initiate [2+2] photocycloaddition of terminal coumarin moieties (Figure 1A). Exploiting the difference in the absorption of coumarin and its photodimer, along with the change in hydrodynamic volume from linear to cyclic form of the allylic sulfide, the reaction was monitored by UV/Vis spectroscopy and size exclusion chromatography (SEC). Coumarin groups absorb light at wavelength from *λ*=300–350 nm with the maxima at *λ*_max_=320 nm while coumarin photodimers show no light absorption in this range.^[15]^ Over the course of 240 mins of UVA irradiation, the absorbance between *λ*=300–350 nm decreased to close to zero (Figure 1B). Using the isosbestic point of the dimerization reaction identified at *λ*=267 nm from the UV/Vis spectra, the change in hydrodynamic radius was monitored via SEC. Before UVA irradiation, the solution contains only linear **C2**, displaying a peak elution time of 28.3 min (Figure 1C). After **C2** was irradiated with UVA for 30 min, a new peak at later elution time (29.1 min) appeared, indicating a more compact hydrodynamic volume of the cyclic monomer **C3**. The intensity of the new peak rises while the intensity of the **C2** peak reduces with prolonged UVA exposure time. Additionally, higher molecular weight shoulders arise at earlier elution times compared to **C2**, indicating intermolecular coumarin dimerization. The intensity of these peaks is significantly lower than the peak of cyclic monomer **C3**, suggesting sufficient selectivity for the intramolecular dimerization at concentrations of 2 mg mL^−1^. After exposure to UVA light for 240 min, the reaction solution showed no characteristic absorbance of the coumarin group and the peak attributable to the linear monomer **C2** was depleted in the SEC trace, indicating complete dimerization. The reaction product was subsequently purified by reversed phase‐HPLC to remove higher molecular weight species and **C3** was obtained in a yield of 73 %. This yield is significantly higher than comparable cyclization of allylic sulfides carried out through dropwise addition of the crosslinkers ranging from 15–25 %.^[14a]^ Since the photocycloaddition must compete with radiative and non‐radiative deactivation processes, higher quantum yields for the intra‐ over intermolecular photocycloaddition enable the observed selectivity for the cyclization.^[16]^

**Figure 1:**
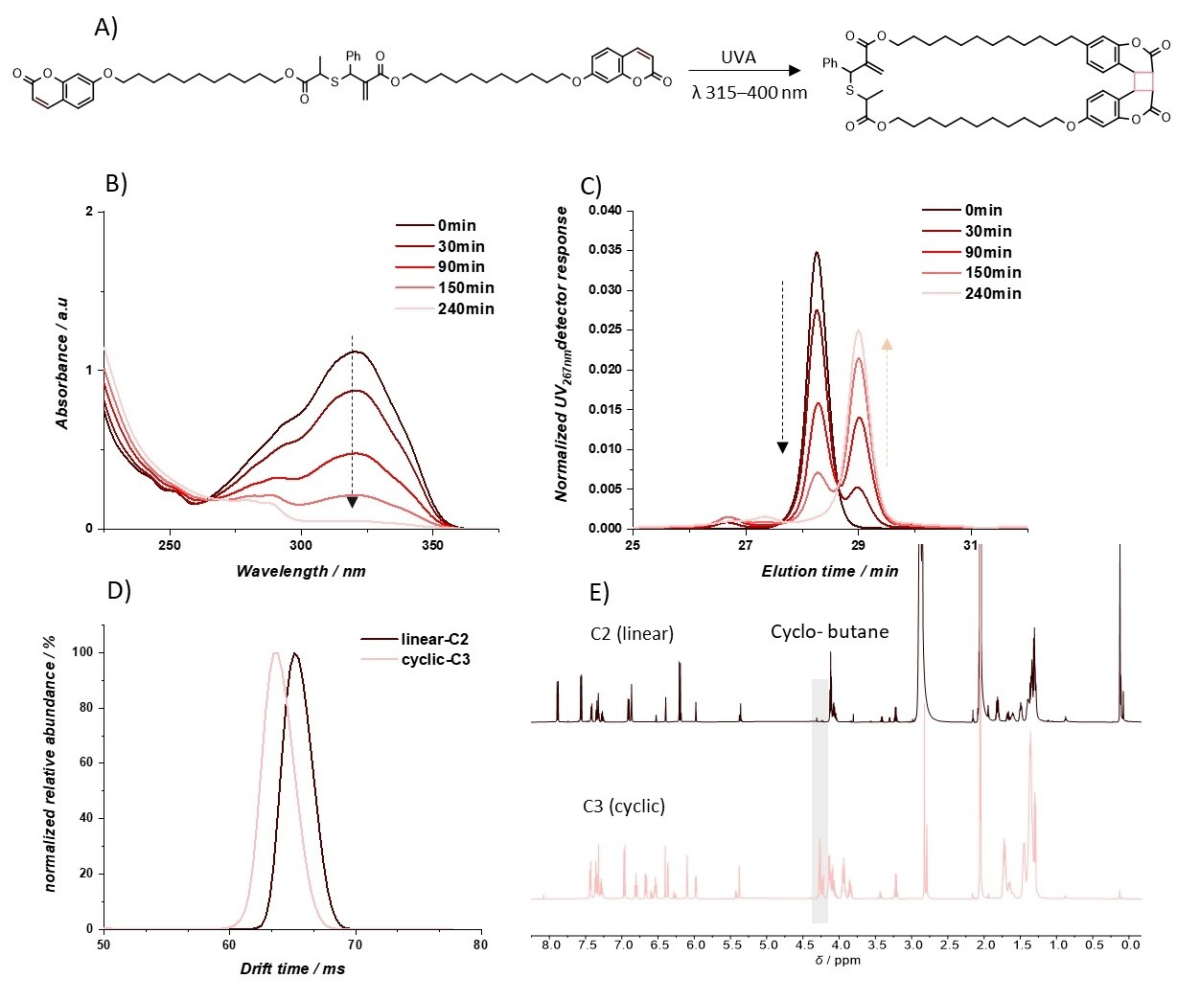
A) Dimerization reaction under UVA to form cyclic monomer (**C3**) from linear analogue (**C2**). B) UV/Vis spectra of diluted reaction solution (0.033 mg mL^−1^ in ACN) over the course of the reaction. C) SEC chromatograms of diluted reaction solution (0.35 mg mL^−1^ in THF) over the course of the reaction. D) Drift time distribution of cyclic monomer and linear analogue after a single pass around the cIM‐MS device. E) ^1^H NMR (acetone‐D6) of cyclic monomer and linear analogue.

The high‐resolution mass spectrum of purified product **C3** (Figure S20) showed an identical *m*/*z* to the linear precursor **C2**, indicative of intramolecular [2+2] photocycloaddition. To further evidence the intramolecular cyclisation, cyclic ion mobility mass spectrometry (cIM‐MS) analysis was used to measure the arrival time of both the linear and the cyclic monomer structures in separate experiments. Mass selected precursor ions at *m*/*z* 917 ([**C2**+Na]^+^ or [**C3**+Na]^+^), were mobility‐separated over a single pass around the cIM‐MS device, with the ionized cyclic monomer having a shorter drift time (*i.e*. smaller collision cross‐section) compared to the linear analogue (Figure 1D). This confirms the morphological transformation from linear monomer **C2** to more compact cyclic monomer **C3** via intramolecular cyclisation of **C2**.

Dimerization of the coumarin groups was further evidenced by the appearance of characteristic aromatic proton resonances in the ^1^H NMR spectrum of the cyclic monomer **C3** (Figure 1E). Two groups of aromatic protons (*δ*=6.96 (2 H), 6.67 (2 H), 6.10 (2 H) ppm and *δ*=6.84–6.76 (2 H), 6.55–6.50 (2 H), 6.36 (2 H)) were found to couple with each other in homonuclear correlation spectroscopy (COSY) of **C3** (Figure S22). Given that the [2+2] cycloaddition of the coumarin groups can potentially form four dimer products,^[15b]^ it is reasonable to assign these proton resonances to two isomers arising from the dimerization reaction (full assignment of NMR spectrum is provided in Figure S21). To verify NMR assignment, multipass cIM‐MS analysis was undertaken.^[17]^ The linear allylic sulfide **C2** consists of 2 diastereomers as evidenced by the resonances of the proton in β‐position to the double bond in the ^1^H NMR spectrum (*δ*=5.36 and 5.38 ppm, Figure S16) and cIM‐MS analysis of **C2** (drift time 765 ms and 773 ms after 31 passes around the cyclic ion mobility device, Figure S18, S19). If each diastereomer undergoes dimerization to afford two isomeric dimers, then the cyclic monomer **C3** should be the mixture of four isomers. Indeed, by employing multiple passes around the cIM‐MS, four isomers were separated with different drift times (Figure S23) but possessing identical underlying mass spectra (Figure S24). In summary, the intramolecular coumarin dimerization of linear monomer **C2** occurred predominantly in the set‐up conditions to give the cyclic monomer **C3**.

The cyclic monomer **C3** was observed to undergo radical ring‐opening polymerization, in which upon radical attack on the monomer double bond, the C−S bonding cleaves to form a sulfur centered propagating radical (Scheme S2). The radical copolymerization of cyclic monomer **C3** and methyl acrylate (MA) monomer ([**C3**]/[MA]=2 %) with 2‐(Dodecylthiocarbonothioylthio) propionic acid as Chain Transfer Agent (CTA, [MA]/[CTA]=200) and azobisisobutyronitrile as initiator (Figure 2A) yielded copolymer **P1** (MA conversion 85 %, **C3** conversion 88 %, Mn=15 200 g mol^−1^, dispersity *Đ*=1.37, Figure S25). Comparing the ^1^H NMR spectra of PMA, cyclic monomer **C3**, and the obtained copolymer, evidence of ring‐opening polymerization of **C3** can be seen (Figure 2B). The resonances of double bond protons in cyclic monomer c_1_ and c_1′_ at *δ*=6.40 and 5.98 ppm deplete in the spectrum of copolymer **P1**. Similarly, the resonance of proton c_2_ of the adjacent carbon at *δ*=5.38 ppm also depletes, whereas a new resonance arises at *δ*=7.72 ppm for the resulting vinylarene of polymer **P1** (Figure 2B, bottom). Using the integral of methyl proton resonance of the MA comonomer, p1, at *δ*=3.66 ppm, the incorporation ratio was calculated from the integrals **p_2_** and **p_1_** and found to be 2 %, equal to the feed ratio. The copolymerization was found to follow pseudo‐first order kinetics, showing a linear increase in ln[Mo]/[Mn] with increased time, as expected for the kinetics of controlled RAFT polymerization (Figure S1).^[18]^ Conversion of vinylic monomer MA and cyclic monomer **C3** remained comparable during the polymerization process and continuously increased up to 120 min polymerization time (Figure 2C), while the copolymer molar weight rose linearly with MA conversion (Figure 2D).

**Figure 2:**
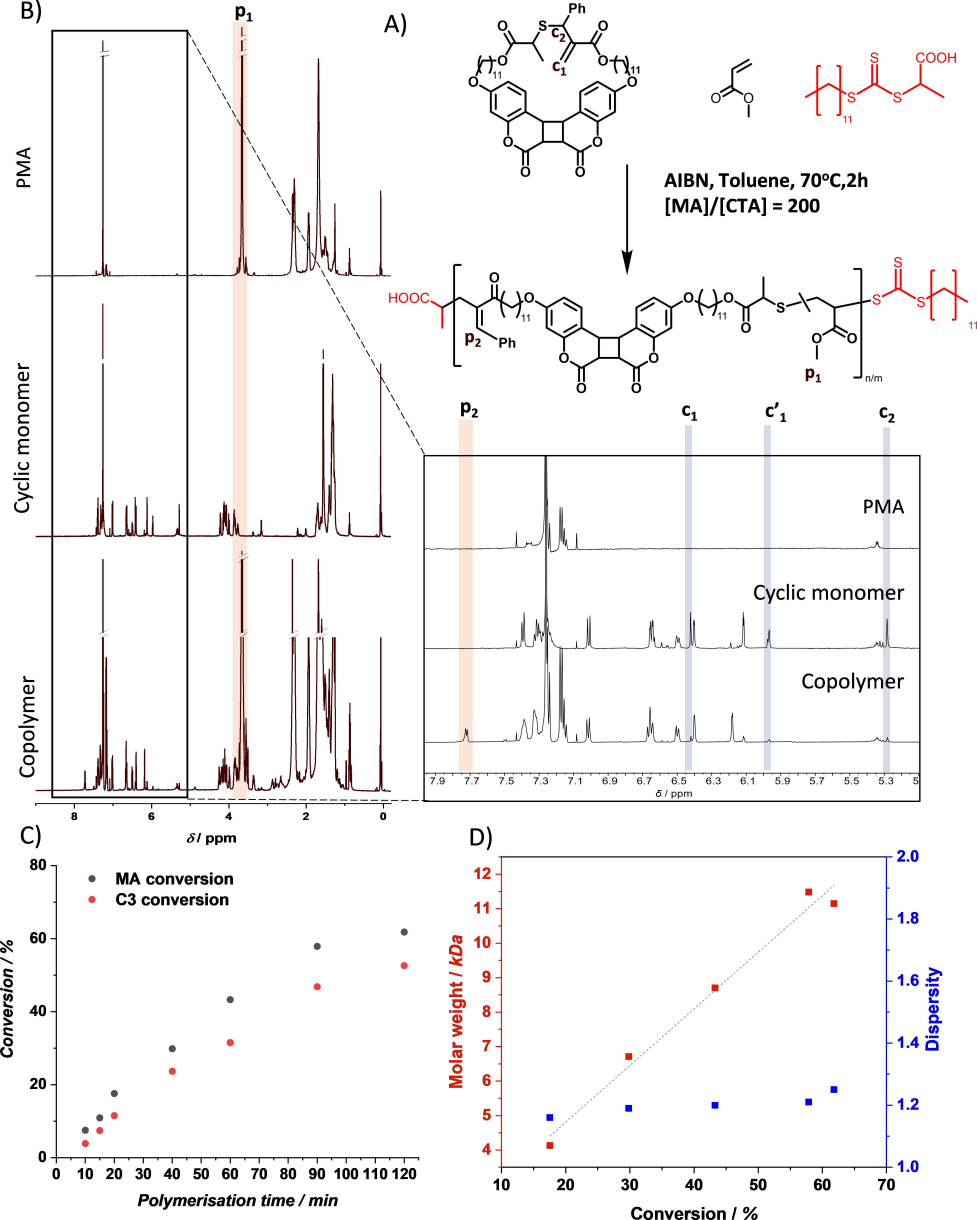
A) RAFT mediated copolymerization reaction of MA and cyclic monomer **C3** to form copolymer **P1**. B) ^1^H NMR (CDCl_3_) of PMA, cyclic monomer **C3**, and copolymer **P1**. C) SEC chromatogram of **P1** showing the mass distribution of the obtained copolymer. C) Plot of MA and cyclic monomer **C3** conversion versus polymerization time. D) Polymer molar weight (red) and dispersity (blue) as a function of MA conversion. Note that the polymers with polymerization time of 10 min and 15 min are not plotted in (D) as their molar weight was too small for discrimination of the polymer and residual **C3** peaks by SEC.

To investigate photodegradation of **P1**, a solution of **P1** in THF (1 mg mL^−1^) in a quartz cuvette was exposed to UVB light in a photoreactor. The degradation of the polymer solution was monitored by SEC (Figure 3B and Table S2). After 5 min of UVB irradiation, the degradation is clearly observed with the molar weight Mn decreased by 34 % (Mn 9900 gmol^−1^, *Đ*=1.47). The molar weight of polymer experiences significant reduction (71 %) after 15 min UVB exposure and decreases further by only 3 % when prolonging the irradiation time to 120 min, suggesting that the photodegradation is complete after 120 min. The degraded polymer had molar weight Mn=4000 g mol^−1^ and *Đ*=1.90. In contrast, when PMA homopolymer **P2** (molar weight Mn=11 600 g mol^−1^, *Đ*=1.37, Figure S26) was irradiated under identical conditions, no signs of degradation were observed via SEC (Figure S5). This indicated that while the RAFT end group is UV sensitive, the photodegradability of the copolymer only stems from the cyclic photolabile monomer **C3**. To probe the proposed ring‐opening polymerization and photodegradation mechanism, the degradation products of **P1** were analyzed via SEC‐HRMS analysis. The mass spectrum (*m*/*z* 1950–2350) obtained at an elution time of 16.0 minutes displays the characteristic mass to charge (*m*/*z*) pattern of triply charged PMA oligomers, with a spacing Δ*m*/*z* 28.68 corresponding to one third mass of methyl acrylate (Figure 3C and 3D), demonstrating that degraded polymer solutions contain MA oligomers. To identify the chemical structure of these oligomers, the *m*/*z* of MA oligomer ions with putative coumarin end groups (shown in Figure 3A) were simulated for comparison. The simulated spectra are in good agreement with the measured spectrum, with mass deviation |Δ*m*|≤1 ppm (Figure S2 and Table S1) indicating that the series of oligomers observed in Figure 3D contain 57–67 MA repeat units. The most abundant *m*/*z* isotope of the resulting oligomers were used to generate extracted ion chromatograms (XICs, Figure 3E) from the SEC‐MS. Oligomers with longer chains elute earlier since the hydrodynamic volume increases with chain length. These results lead to the conclusion that the photodegradability of the copolymer **P1** is inherited from the cyclic monomers, and that the photodegradation occurs via the photocycloreversion of the backbone imbedded coumarin dimers, i.e. the photo‐labile linkers.

**Figure 3:**
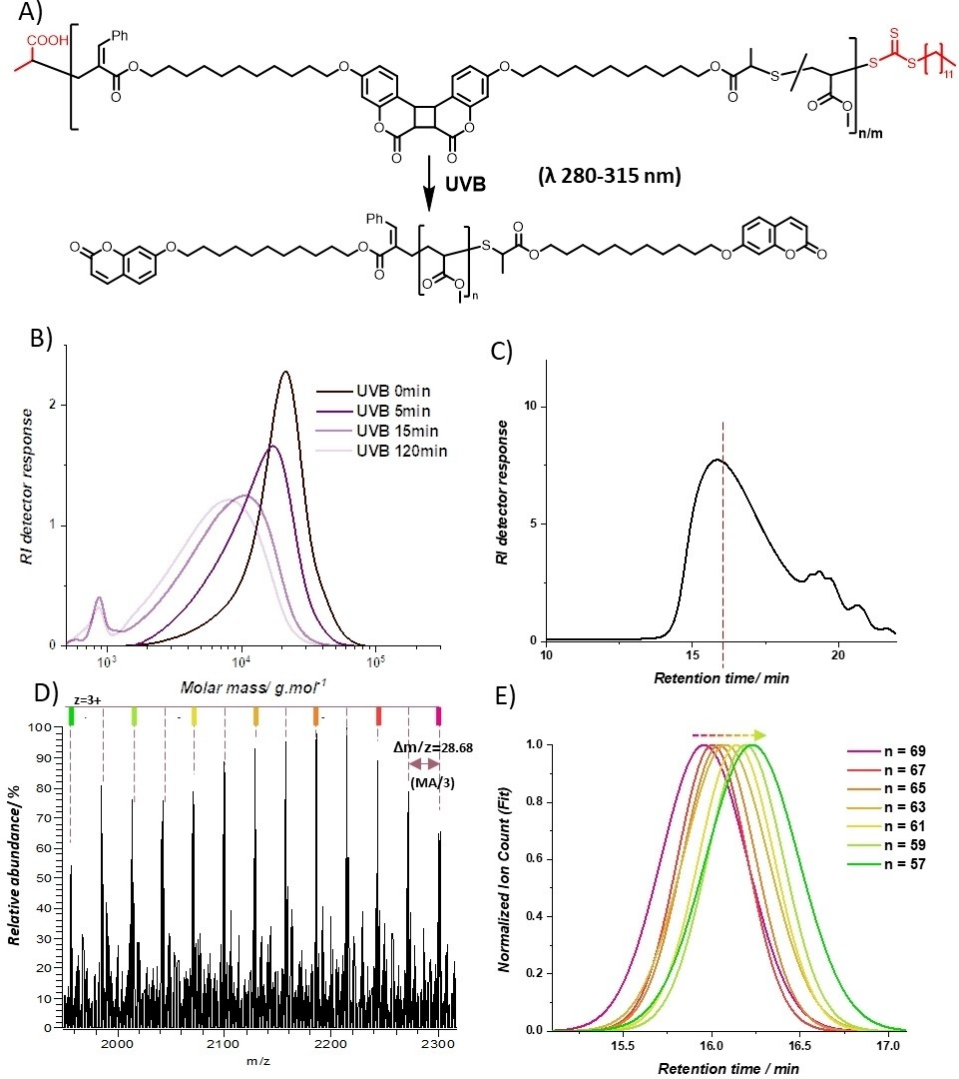
A) depolymerization reaction of the copolymer under UVB light. B) SEC chromatograms showing mass distribution of the copolymer during UVB exposure. C) SEC chromatogram of SEC‐ESI‐MS results of the 120 min UVB‐irradiated copolymer. D) Mass spectra at elution time 16.0 min, *m*/*z* 1950–2350, with peaks assigned for corresponding oligomer ions in panel E. E) Fitted XIC traces of triply charged sodiated oligomer ions (number of MA unit=57–69) extracted from the SEC‐ESI‐MS.

The photodegradability of copolymers having different molar mass and incorporation ratio of photolabile groups into the polymer was also examined. The polymers prepared with polymerization times of 40 min, 60 min, 90 min and 120 min (**P4–P7**) from the kinetics experiment (section 4.2.2 in Supporting Information, Table S3), having different molar mass but comparable ratio of photoresponsive group in the polymer chain (1.7 %), were exposed to UVB irradiation. After 30 min under UVB irradiation, all polymers showed significant change in molar mass distribution (Figure S3), with the reduction in molar weight of polymer after photodegradation tracking with increased polymer Mn (Table S3). Raising the [MA]/[CTA] ratio to 400 and reducing feed ratio of [**C3**]/[MA] to 1 % yielded the copolymer **P3** (MA conversion 86 %, **C3** conversion 88 %, incorporation ration 0.8 %, Mn=26.8 kg mol^−1^, dispersity *Ð*=1.33). **P3** showed a 63 % decrease in Mn after 30 min UVB exposure. Irradiating **P3** for a further 30 min did not lead to any further reduction in Mn, indicating the complete degradation after 30 min UVB irradiation (Table S4, Figure S4).

The ability to degrade under real world conditions was investigated by exposing a quartz cuvette containing a solution of 0.7 mg mL^−1^ copolymer in THF to sunlight for 10 days, with a control sample of identical concentration stored in lab conditions away from natural light exposure. Changes in mass distribution of the polymer were monitored by SEC over the duration of the experiment. The intensity at high mass range exhibits a significant decrease with prolonged sunlight exposure and the peak clearly shifts to lower mass (Figure 4), while the control experiment showed no change in mass distribution (Figure S6), indicating that natural sunlight exposure is capable of triggering photodegradation of the polymer.

**Figure 4:**
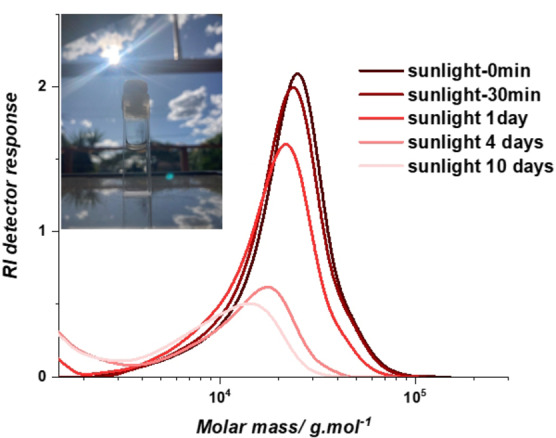
SEC chromatograms showing mass distribution of the copolymer solution following solar irradiation for 0 min, 30 min, 1 day, 4 days and 10 days.

In summary, we report a synthetic strategy to program inherent photodegradability into vinylic polymers based on ring‐opening polymerization of a cyclic monomer bearing a photolabile group. The macrocyclic monomer **C3** was synthetically accessible through intramolecular [2+2] photocycloaddition of coumarin terminated allylsulfide **C2**. Upon RAFT mediated copolymerization of MA with the cyclic monomer **C3**, the cyclobutane moieties of **C3** were imbedded into the resulting polymer **P1** through radical ring‐opening polymerization of **C3**. Exposure of **P1** to UVB light for 2 h induced photocycloreversion of the cyclobutane moieties within the polymer backbone leading to scission of the polymer main chain. While the polymer was stable in the laboratory, degradation in the presence of sunlight was observed after 30 min and significant degradation was achieved after 10 days. Coumarin was chosen as the photoreactive group for the photoreversible cycloaddition controlling monomer formation and polymer degradation because the high energy required for cycloreversion enabled sufficient bench stability for analytical characterization. However, an extensive toolbox of orthogonal dienes has been developed over the last years, with a wide range of [2+2] photocycloaddition and –reversion wavelengths.^[19]^ The modular nature of the developed technique herein paves the way to integrating an array of photocycloaddition products into polymer backbones of vinylic monomers, giving rise to polymers with freely tunable degradation wavelengths.

## Conflict of interest

The authors declare no conflict of interest.

## Supporting information

As a service to our authors and readers, this journal provides supporting information supplied by the authors. Such materials are peer reviewed and may be re‐organized for online delivery, but are not copy‐edited or typeset. Technical support issues arising from supporting information (other than missing files) should be addressed to the authors.

Supporting Information

## Data Availability

The data that support the findings of this study are available in the Supporting Information of this article.

## References

[1] M. Wei , Y. Gao , X. Li , M. J. Serpe , Polym. Chem. 2017, 8, 127–143.

[2a] C. Zhu , C. Ninh , C. J. Bettinger , Biomacromolecules 2014, 15, 3474–3494; 25226507 10.1021/bm500990z

[2b] O. Bertrand , J.-F. Gohy , Polym. Chem. 2017, 8, 52–73.

[3a] A. B. Samarasekara, A. Anuradha, K. De Zoysa, *Proceedings of International Forestry and Environment Symposium*, *Vol. 17*, **2012**;

[3b] I. Kyrikou , D. Briassoulis , J. Polym. Environ. 2007, 15, 125–150;

[3c] B. C. Daglen , D. R. Tyler , Green Chem. Lett. Rev. 2010, 3, 69–82.

[4a] J. Jiang , X. Tong , D. Morris , Y. Zhao , Macromolecules 2006, 39, 4633–4640;

[4b] E. Cabane , V. Malinova , W. Meier , Macromol. Chem. Phys. 2010, 211, 1847–1856;

[4c] G. Liu , X. Wang , J. Hu , G. Zhang , S. Liu , J. Am. Chem. Soc. 2014, 136, 7492–7497. 24786176 10.1021/ja5030832

[5a] L. Ionov , S. Diez , J. Am. Chem. Soc. 2009, 131, 13315–13319; 19711979 10.1021/ja902660s

[5b] C. A. DeForest , K. S. Anseth , Angew. Chem. Int. Ed. 2012, 51, 1816–1819; 10.1002/anie.201106463 PMC3430005 22162285

[5c] V. X. Truong , K. M. Tsang , G. P. Simon , R. L. Boyd , R. A. Evans , H. Thissen , J. S. Forsythe , Biomacromolecules 2015, 16, 2246–2253. 26056855 10.1021/acs.biomac.5b00706

[6a] A. Sommazzi , F. Garbassi , Prog. Polym. Sci. 1997, 22, 1547–1605;

[6b] G. H. Hartley , J. Guillet , Macromolecules 1968, 1, 165–170.

[7a] C. Petit , J. Bachmann , L. Michalek , Y. Catel , E. Blasco , J. P. Blinco , A.-N. Unterreiner , C. Barner-Kowollik , Chem. Commun. 2021, 57, 2911–2914; 10.1039/d1cc00124h 33616594

[7b] D. Han , X. Tong , Y. Zhao , Macromolecules 2011, 44, 437–439.

[8] S. Sun , E. A. Chamsaz , A. Joy , ACS Macro Lett. 2012, 1, 1184–1188. 35607192 10.1021/mz3002947

[9] G. Pasparakis , T. Manouras , A. Selimis , M. Vamvakaki , P. Argitis , Angew. Chem. Int. Ed. 2011, 50, 4142–4145; 10.1002/anie.201007310 21433230

[10] H. Frisch , K. Mundsinger , B. L. J. Poad , S. J. Blanksby , C. Barner-Kowollik , Chem. Sci. 2020, 11, 2834–2842. 32206267 10.1039/c9sc05381f PMC7069517

[11] P. Johnston , C. Braybrook , K. Saito , Chem. Sci. 2012, 3, 2301–2306.

[12a] P. Nesvadba , Encyclopedia of Radicals in Chemistry, Biology and Materials, Wiley, Hoboken, 2012, p. 3;

[12b] N. Corrigan , K. Jung , G. Moad , C. J. Hawker , K. Matyjaszewski , C. Boyer , Prog. Polym. Sci. 2020, 111, 101311.

[13a] A. Tardy , J. Nicolas , D. Gigmes , C. Lefay , Y. Guillaneuf , Chem. Rev. 2017, 117, 1319–1406; 28085265 10.1021/acs.chemrev.6b00319

[13b] T. Pesenti , J. Nicolas , ACS Macro Lett. 2020, 9, 1812–1835; 35653672 10.1021/acsmacrolett.0c00676

[13c] W. J. Bailey , Polym. J. 1985, 17, 85–95;

[13d] F. Sanda , T. Endo , J. Polym. Sci. Part A 2001, 39, 265–276.

[14a] J. M. Paulusse , R. J. Amir , R. A. Evans , C. J. Hawker , J. Am. Chem. Soc. 2009, 131, 9805–9812; 19555103 10.1021/ja903245p PMC2753401

[14b] H. Huang , B. Sun , Y. Huang , J. Niu , J. Am. Chem. Soc. 2018, 140, 10402–10406; 29920082 10.1021/jacs.8b05365

[14c] W. Wang , Z. Zhou , D. Sathe , X. Tang , S. Moran , J. Jin , F. Haeffner , J. Wang , J. Niu , Angew. Chem. Int. Ed. 2022, 61, e202113302; 10.1002/anie.202113302 34890493

[15a] J. He , L. Tremblay , S. Lacelle , Y. Zhao , Soft Matter 2011, 7, 2380–2386;

[15b] X. Yu , D. Scheller , O. Rademacher , T. Wolff , J. Org. Chem. 2003, 68, 7386–7399. 12968891 10.1021/jo034627m

[16] H. Frisch , J. P. Menzel , F. R. Bloesser , D. E. Marschner , K. Mundsinger , C. Barner-Kowollik , J. Am. Chem. Soc. 2018, 140, 9551–9557. 29965750 10.1021/jacs.8b04531

[17a] D. G. Delafield , G. Lu , C. J. Kaminsky , L. Li , TrAC Trends Anal. Chem. 2022, 157, 116761;

[17b] K. Giles , J. Ujma , J. Wildgoose , S. Pringle , K. Richardson , D. Langridge , M. Green , Anal. Chem. 2019, 91, 8564–8573. 31141659 10.1021/acs.analchem.9b01838

[18] A. Goto , T. Fukuda , Prog. Polym. Sci. 2004, 29, 329–385.

[19a] T. Doi , H. Kawai , K. Murayama , H. Kashida , H. Asanuma , Chem. Eur. J. 2016, 22, 10533–10538; 27299696 10.1002/chem.201602006

[19b] K. Kalayci , H. Frisch , V. X. Truong , C. Barner-Kowollik , Nat. Commun. 2020, 11, 4193; 32826921 10.1038/s41467-020-18057-9 PMC7443129

[19c] S. Ludwanowski , D. Hoenders , K. Kalayci , H. Frisch , C. Barner-Kowollik , A. Walther , Chem. Commun. 2021, 57, 805–808; 10.1039/d0cc07429b 33367359

[19d] K. Kalayci , H. Frisch , C. Barner-Kowollik , V. X. Truong , Adv. Funct. Mater. 2020, 30, 1908171;

[19e] D. Kodura , L. L. Rodrigues , S. L. Walden , A. S. Goldmann , H. Frisch , C. Barner-Kowollik , J. Am. Chem. Soc. 2022, 144, 6343–6348. 35364816 10.1021/jacs.2c00156

