# Supplementary material for: Programming Photodegradability into Vinylic Polymers via Radical Ring‐Opening Polymerization
Source: Angew Chem Int Ed Engl. 2023 Jan 9;62(6):e202213511. doi: 10.1002/anie.202213511 (PMC10108003; doi:10.1002/anie.202213511)
Supplement: Supplementary file 1 — Supporting Information [file ANIE-62-0-s001.pdf]

Angewandte Chemie
Eine Zeitschrift der Gesellschaft Deutscher Chemiker

Supporting Information

Programming Photodegradability into Vinylic Polymers via Radical Ring-Opening Polymerization

P. T. Do, B. L. J. Poad, H. Frisch**

Supporting Information

Programming Photodegradability into Vinylic Polymers via Radical Ring-opening Polymerisation

Phuong T. Do,[1,2] Berwyck L. J. Poad, [1,2,3]* Hendrik Frisch[1,2]*

[1] School of Chemistry and Physics, Queensland University of Technology (QUT), 2 George Street, Brisbane, QLD 4000, Australia.

[2] Centre for Materials Science, Queensland University of Technology (QUT), 2 George Street, Brisbane, QLD 4000, Australia.

[3] Mass Spectrometry Development Laboratory, Central Analytical Research Facility, Queensland University of Technology (QUT), 2 George Street, Brisbane, QLD 4000, Australia.

Contents

1. Additional data 3
2. Experimental details 8
2.1. SEC-ESI-MS 8
2.2. LC-ESI-MS 8
2.3. IM-MS 8
2.4. THF-SEC 8
2.5. 1D NMR Measurements 9
2.6. UV-VIS Spectroscopy 9
2.7. Degradation under UVB light 9
2.8. Degradation under sunlight 9
3. Synthesis procedure of cyclic macro monomer: 9
3.1. Materials 9
3.2. Synthesis 10
3.2.1. S1 10
3.2.2. S2 11
3.2.3. S3 12
3.2.4. S4 13
3.2.5. C1 15
3.2.6. C2 17
3.2.7. C3 20
4. Polymer synthesis 24
4.1. Material 24
4.2. Synthesis 24
4.2.1. Synthesis of polymer 24
4.2.2. Kinetic study of the copolymerisation 26

1. Additional data

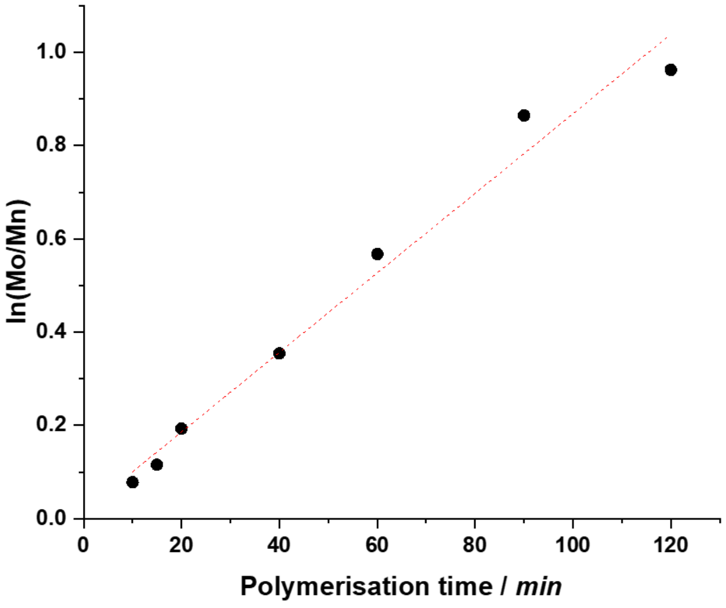

Figure S 1. Plot of ln(Mo/Mn) versus polymerisation time of copolymerisation of cyclic monomer C3 and methyl acrylate MA with [C3]/[MA]=2%. See section 4.2.2 for more information.

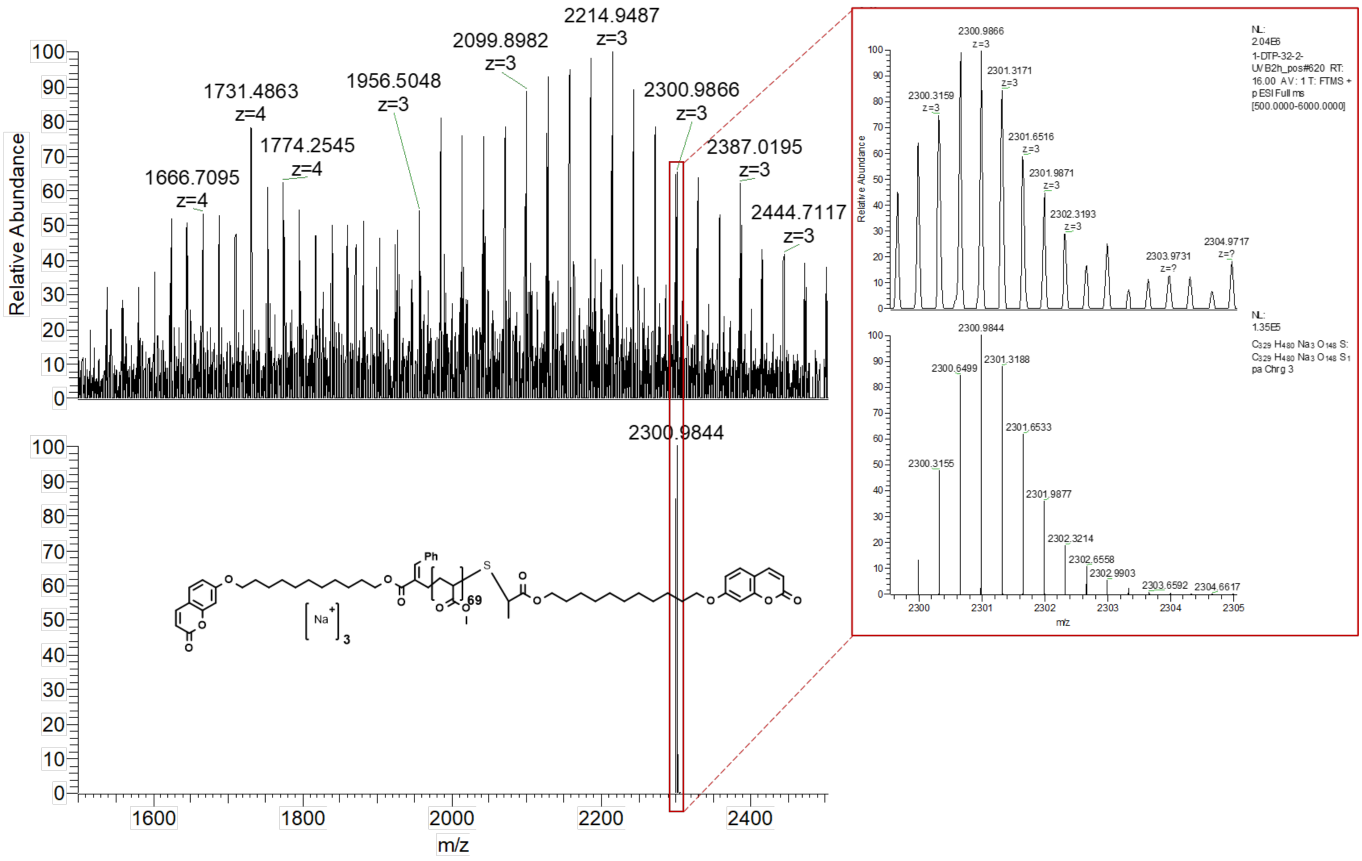

Figure S 2. Mass spectrum at retention time 16.0 min (upper) and the simulated for triply charged sodiated oligomer ions with 69 units of MA $C_{329}H_{480}Na_3O_{148}S^{3+}$ (lower) (simulated m/z: 2300.9844, measured m/z: 2300.9866, Δm 0.96ppm). Inset is the zoom in mass range showing isotopic patterns of the ion.

number of MA units in oligomer	*m/z* simulated (most abundant isotopic feature)	*m/z* found	Δ*m* (ppm)
69	2300.9844	2300.9866	0.96
68	2272.3055	2272.3057	0.09
67	2243.6266	2243.6257	-0.4
66	2214.9476	2214.9487	0.5
65	2186.2687	2186.2686	-0.05
64	2157.5898	2157.5903	0.23
63	2128.9108	2128.9116	0.38
62	2100.2319	2100.2312	-0.33
61	2071.553	2071.5518	-0.58
60	2042.8741	2042.8752	0.54
59	2014.1951	2014.1962	0.55
58	1985.5162	1985.5149	-0.65
57	1956.8373	1956.839	0.87

*Table S 1. Table representing simulated mass to charge ratio m/z (the most abundant peak) of methyl acrylate oligomers with different numbers of MA repeat units as depicted in figure S1 (bottom) and m/z found in the actual mass spectrum of UVB irradiated **P1** solution, and their mass deviation.*

UVB exposure time	Molar weight Mn (kg.mol^{-1})	Dispersity *Đ*	molar weight decrease (%)
0min	15.2	1.37	0
5min	9.9	1.47	34
15min	4.5	2	71
120min	4.0	1.9	74

*Table S 2. Table representing the change in molar weight Mn and dispersity Đ of copolymer **P1** during UVB degradation (section 2.7), identified by SEC.*

polymer	Incorporation ratio of C3 into copolymer	Molar weight Mn before degradation (kg.mol^{-1})	Molar weight Mn after degradation (kg.mol^{-1})	molar weight decrease (%)
**P4**	1.7%	6.7	4.9	27
**P5**	1.7%	8.7	5.7	34
**P6**	1.4%	11.5	6.6	43
**P7**	1.8%	11.2	6.3	44

*Table S 3. Table representing the change in molar weight Mn of copolymer **P4-P7** after UVB irradiation for 30 min (section 2.7), identified by SEC.*

UVB exposure time	Molar weight Mn (kg.mol^{-1})	Dispersity *Đ*	molar weight decrease (%)
0 min	26.8	1.33	0
30min	10	1.59	63
60min	9.8	1.62	63

*Table S 4. Table representing the change in molar weight Mn and dispersity Đ of copolymer **P3** during UVB degradation (section 2.7), identified by SEC.*

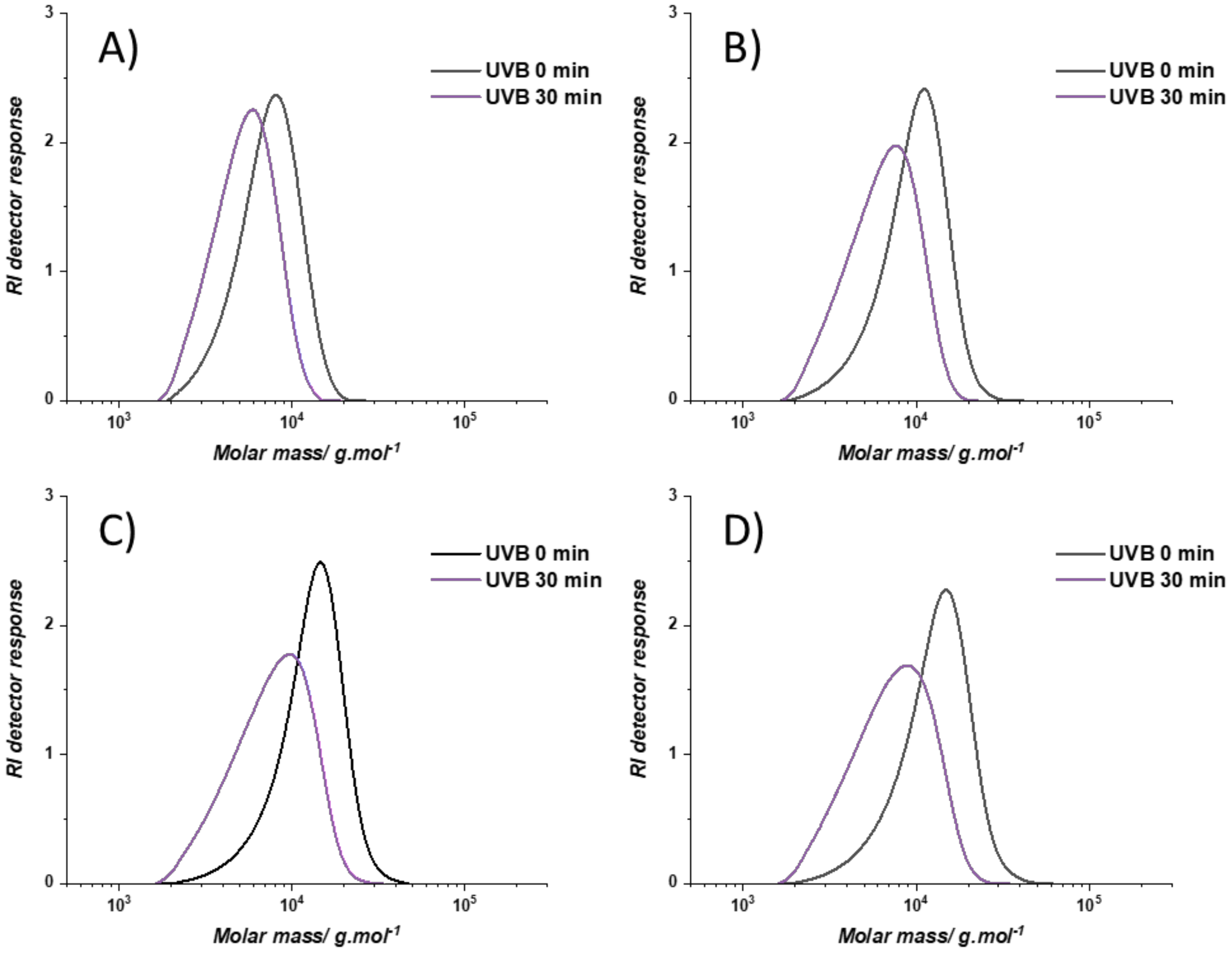

*Figure S 3. SEC trace of copolymer **P4** (A), **P5** (B), **P6** (C), **P7** (D) in THF (1mg mL^{-1}) before and after UVB irradiation for 30 min.*

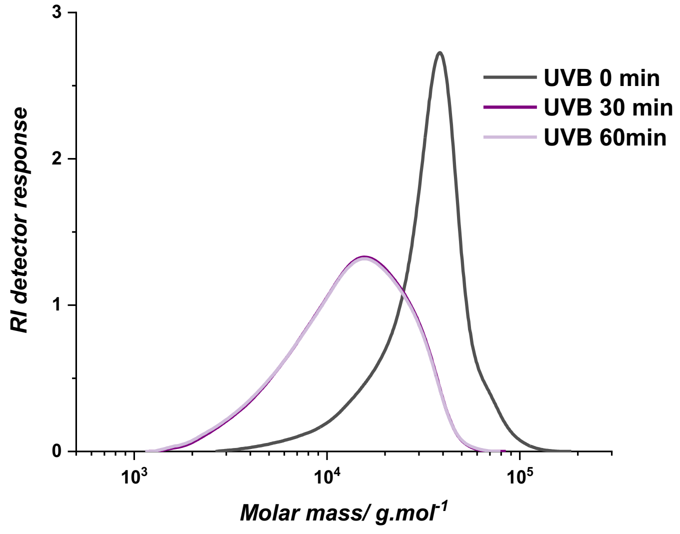

*Figure S 4. SEC trace of copolymer **P3** in THF (1mg mL^{-1}) during UVB photodegradation.*

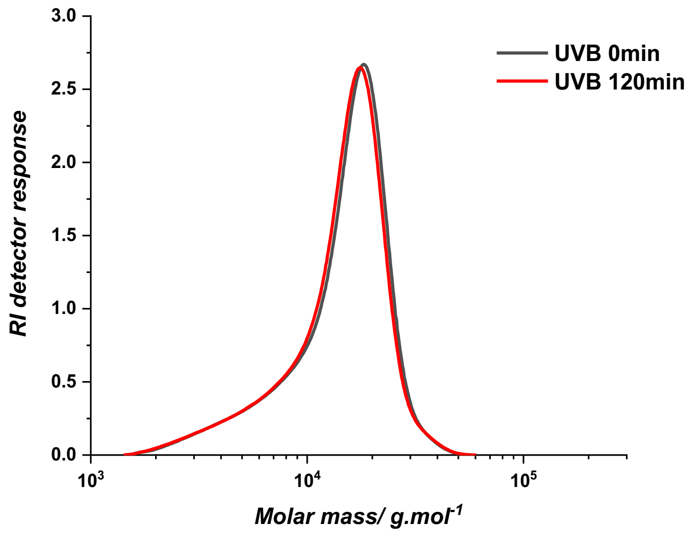

*Figure S 5. SEC of PMA homopolymer **P2** in THF (1mg mL^{-1}) before and after irradiation with UVB light for 120 min.*

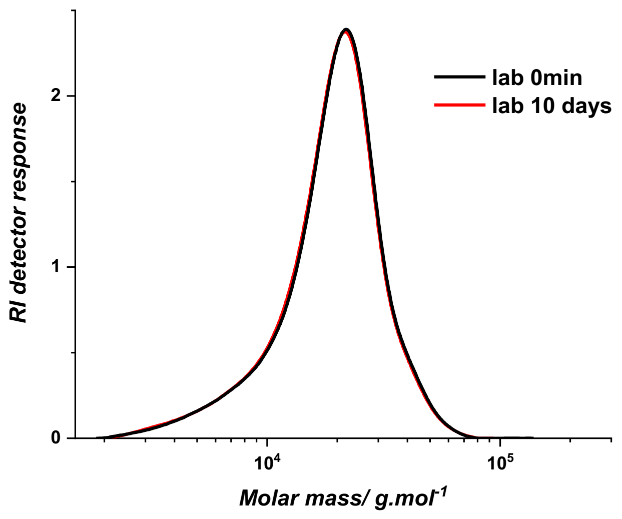

*Figure S 6. SEC of copolymer **P1** in THF (0.7mg mL$^{-1)}$ directly after solution preparation and after the solution was stored at ambient conditions open to laboratory light in the lab for 10 days.*

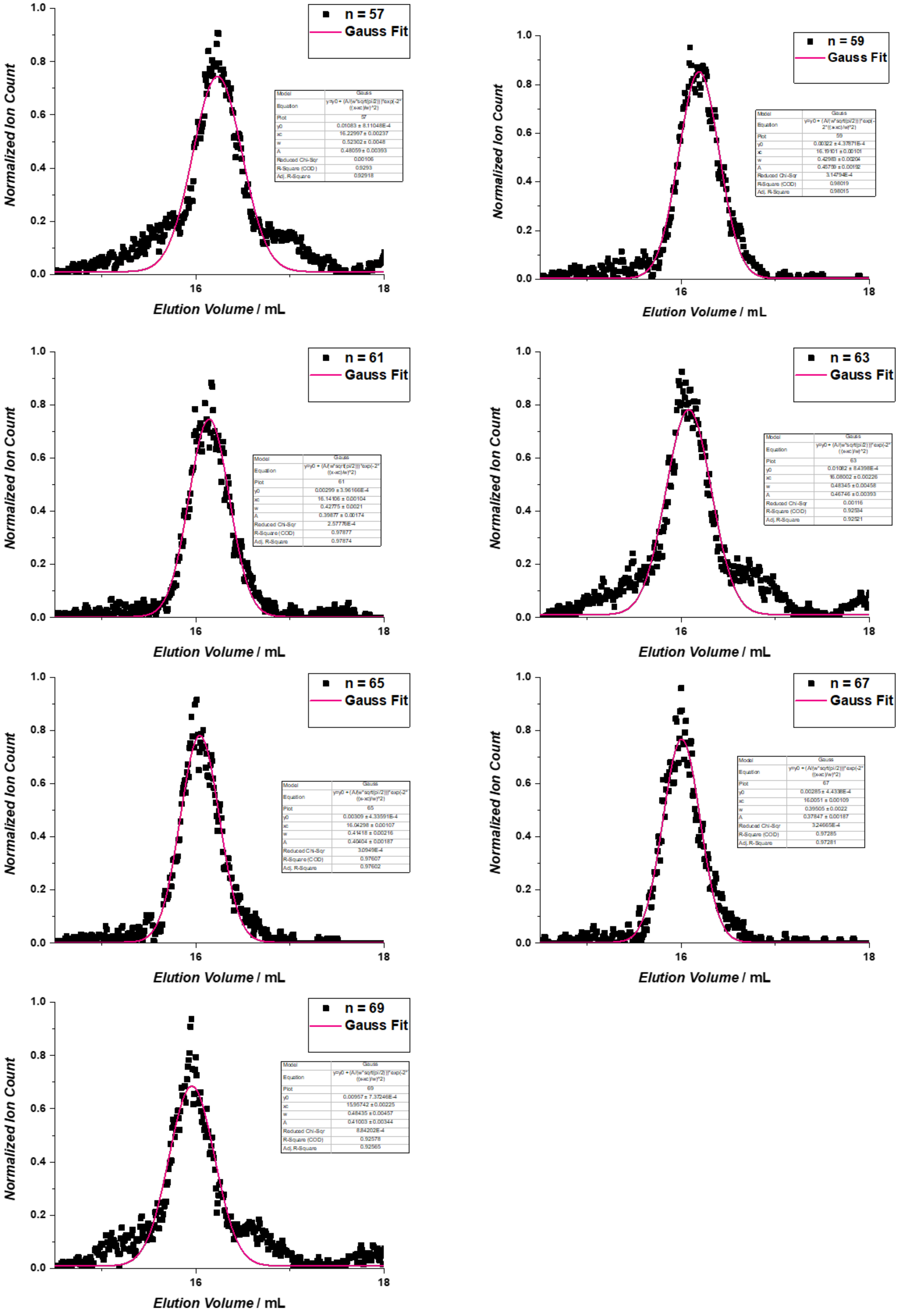

Figure S 7. Gaussian fit (magenta line) for individual XIC traces (black dots) extracted from SEC-MS of UVB degraded polymer solution for the oligomers with number of MA repeating units from 57-69. The combination of these fitted XIC traces is represented in Figure 3D in the main paper.

2. Experimental details

2.1. SEC-ESI-MS

Size exclusion chromatography coupled with electrospray ionisation mass spectrometry experiments were measured using a Q Exactive Plus Biopharma mass spectrometer (Thermo Fisher Scientific, San Jose, CA, USA) equipped with a HESI-II ionisation source. The mass spectrometer was calibrated up to *m/z* 2000 using premixed calibration solution (Pierce; Thermo Scientific) and for high mass mode (*m/z* 600-8000) using ammonium hexafluorophosphate solution. A constant spray voltage of +3.5 kV, a dimensionless sheath gas and a dimensionless auxiliary gas flow rate of 10 and 0 were applied, respectively. The capillary temperature was set to 320 °C, the S-lens RF level was set to 150 and the auxiliary gas heater temperature was set to 125 °C. The Q Exactive was coupled to an UltiMate 3000 UHPLC System (Dionex, Sunnyvale, CA, USA) consisting of a pump (LPG 3400SD), autosampler (WPS 3000TSL), and a temperature-controlled column department (TCC 3000). Separation was performed on two mixed bed size exclusion chromatography columns (Agilent, Mesopore 250 × 4.6 mm, particle diameter 3 µm) with a precolumn (Mesopore 50 × 7.5 mm) operating at 30 °C. THF at a flow rate of 0.30 mL min^{-1} was used as eluent. The mass spectrometer was coupled to the column in parallel with an UV-detector (VWD 3400, Dionex) and a Refractive Index detector (RefractoMax520, ERC, Japan) as described earlier.[1] A split flow of 0.27 mL min^{-1} of the eluent were directed through the UV- and RI-detector and the remaining 30 µL min^{-1} directed into the electrospray source following post-column addition of 50 µM sodium iodide in methanol at 20 µL min^{-1} by a micro-flow HPLC syringe pump (Teledyne ISCO, Model 100DM). A 100 µL aliquot of polymer solution at 2 mg mL^{-1} concentration was injected into the SEC system for analysis.

2.2. LC-ESI-MS

LC-MS measurements were performed on an UltiMate 3000 UHPLC system (Dionex, Sunnyvale, CA, USA) consisting of a pump (LPG 3400SZ, autosampler WPS 3000TSL) and a temperature-controlled column department (TCC 3000). Separation was performed on a C18 HPLC-column (Phenomenex Luna 5µm, 100 Å, 250 × 2.0 mm) held at 40 °C. Mobile phase A was acetonitrile containing 10 mM ammonium acetate, and mobile phase B was water containing 10 mM ammonium acetate. A 15 min linear elution gradient of A:B 10:90 – 80:20 was used at a flow rate of 0.20 mL min^{-1}. The flow was split in a 9:1 ratio, where 90% (0.18 mL min^{-1}) of the eluent was directed through a UV-detector (VWD 3400, Dionex, detector wavelengths 215, 254, 280, 360 nm) and 10% (0.02 mL min^{-1}) was directed to the electrospray source. Spectra were recorded on the same Q Exactive Plus Biopharma mass spectrometer as described above for the SEC-ESI-MS experiments, operating in positive ion mode. A constant spray voltage of 3.0 kV, a dimensionless sheath gas and a dimensionless auxiliary gas flow rate of 25 and 10 were applied, respectively. The capillary temperature was set to 320 °C, the S-lens RF level was set to 60, and the aux gas heater temperature was set to 100 °C.

2.3. IM-MS

Ion mobility mass spectrometry analyses were conducted on an ion mobility enabled quadrupole time of flight cyclic ion mobility mass spectrometer (SELECT SERIES Cyclic IMS; Waters Corporation, Wilmslow, UK) with an associated control and analysis software. The working solution was prepared from stock solution of analyte sample 0.05 mg mL^{-1} and methanolic solution of sodium acetate 0.2mM with the volume ratio of 10:2. The working solution was then infused directly to the MS system at constant flow rate of 5 µL min^{-1} via a 500 µL syringe controlled by a syringe pump (Pump 11 Elite, Harvard Apparatus). The MS was operated in positive-ion mode with operational parameters: capillary voltage, +1 kV; cone voltage, 40 V; desolvation gas flow rate, 800 L/hour; desolvation gas temperature, 250°C; source temperature, 120°C; and Helium cell pressure, 2.3 mbar, cIM cell pressure 1.7 mBar. The precursor ions were selected by a quadrupole, following by ion mobility differentiation in cyclic ion mobility device. The IM travelling wave amplitude and velocity were kept constant at 22.0 V and 375ms^{-1}, respectively, while the number of passes that precursor ions travel around cyclic device varied on experiments. The term "pass" refers to one complete transit of ions around the cyclic ion mobility device. The mass spectra were recorded in the m/z range 50-2000. Data was visualised and analysed using MassLynx (version 4.2; Waters Corporation).

2.4. THF-SEC

The SEC measurements were conducted on a PSS SECurity[2] system consisting of a PSS SECurity Degasser, PSS SECurity TCC6000 Column Oven (35 °C), PSS SDV Column Set (8 x 150 mm 5 µm Precolumn, 8 x 300 mm 5 µm Analytical Columns, 100000 Å, 1000 Å and 100 Å) and an Agilent 1260 Infinity Isocratic Pump, Agilent 1260 Infinity Standard Autosampler, Agilent 1260 Infinity Diode Array and Multiple Wavelength Detector (A: 254 nm, B: 360

nm), Agilent 1260 Infinity Refractive Index Detector (35 °C). HPLC grade THF, stabilized with BHT, is used as eluent at a flow rate of 1 mL·min^{-1}. Narrow disperse linear poly (methyl methacrylate) ($\mathcal{M}_n$: 202 g·mol^{-1} to 2.2x106 g·mol^{-1}) standards (PSS ReadyCal) were used as calibrants. All samples were passed over 0.22 µm PTFE membrane filters. Molecular weight and dispersity analysis was performed in PSS WinGPC UniChrom software (version 8.2).

2.5. 1D NMR Measurements

^{1}H- and COSY spectra were recorded on a Bruker System 600 Ascend LH, equipped with a BBO-Probe (5 mm) with z-gradient (1H: 600.13 MHz, 13C: 150.90 MHz,). All measurements were carried out in deuterated solvents. The chemical shift (δ) is recorded in parts per million (ppm) and relative to the residual solvent protons.[2] The measured coupling constants were calculated in Hertz (Hz). To analyze the spectra the software MESTRENOVA 11.0 was used. The signals were quoted as follows: s = singlet, bs = broad singlet, d = doublet, t = triplet, dd = doublet of doublets and m = multiplet.

2.6. UV-VIS Spectroscopy

UV/vis spectra were recorded on a Shimadzu UV-2700 spectrophotometer equipped with a CPS-100 electronic temperature control cell positioner. Samples were prepared in THF and measured in Hellma Analytics quartz high precision cells with a path length of 10 mm at ambient temperature.

2.7. Degradation under UVB light

A quartz cuvette containing a solution of polymer in THF solvent (1 mg. mL^{-1}) was placed in a Luzchem photoreactor equipped with UVB lamps (λ 280-315 nm). The cuvette was irradiated with UVB light with stirring for certain amount of time which is specified in the result section.

2.8. Degradation under sunlight

A quartz cuvette containing a solution of polymer in THF solvent (0.7 mg mL^{-1}) was placed under the sun in Brisbane, Queensland, Australia for a number of days. The experiment was conducted in the period from 4th of August 2022 to 29th of August 2022. Solar spectra were measured at the same site as the cuvette was placed on the 4th of August 2022 using a portable fibre coupled spectrometer (Flame S-UV-VIS-ES, Ocean Optics).

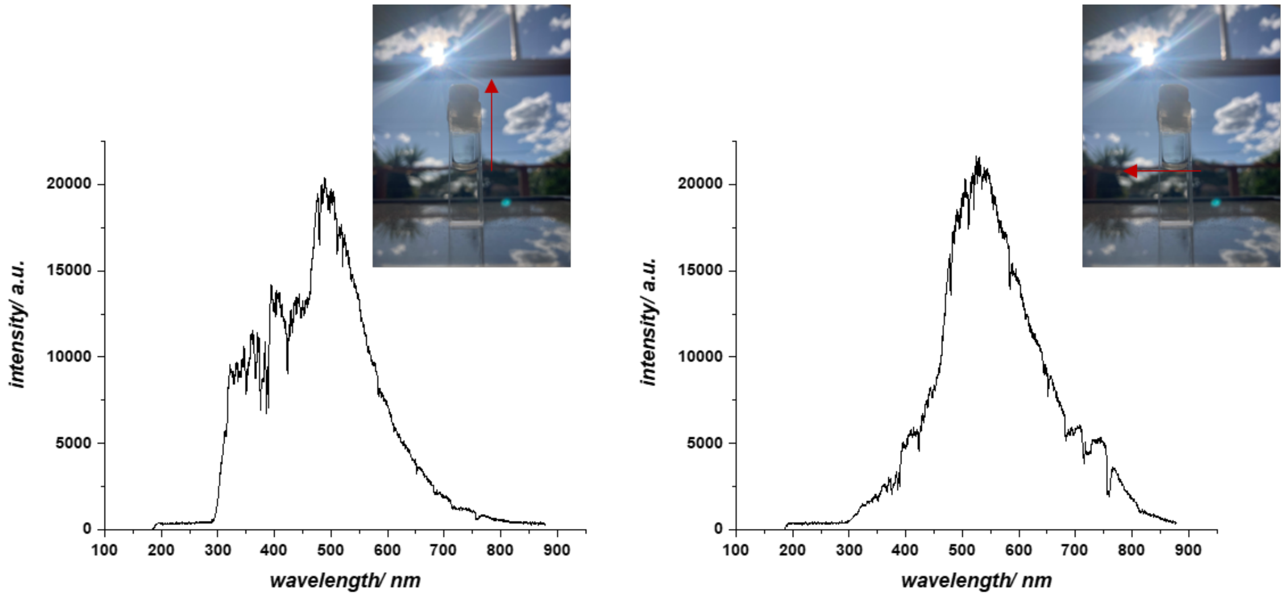

Figure S 8. Solar spectra measured on the first day of the experiments (13:00 4th of August 2022) next to the cuvettes, with the fibre optic tip oriented vertically (left) and horizontally (right) as indicated by the red arrow.

3. Synthesis procedure of cyclic macro monomer:

3.1. Materials

Unless stated otherwise, all chemicals and solvents were used as received from the suppliers without further purification.

Benzaldehyde (Sigma- Aldrich, 99.0% min), tert-butyl acrylate (Sigma-Aldrich, 98.0% min), 1,8-Diazabicyclo [5.4.0] undec-7-ene (Sigma- Aldrich, 98.0% min), hydrochloric acid (32% in water, Thermo Fisher Scientific), sodium sulfate

(anhydrous, granular, Thermo Fisher Scientific, 99.0% min), 4-methoxyphenol (Sigma-Aldrich, 99.0% min), acetic anhydride (Chem-Supply , 99.0% min), N, N-dimethylaminoprydine (Sigma-Aldrich, 99.0%), dichloromethane (Chem-Supply, 99.8%), ethyl acetate (Thermo Fisher Scientific, 99.5%), cyclohexane (Sigma-Aldrich, 99.5%), 1,4-Diazabicyclo [2.2.2] octane (Sigma-Aldrich, 99.0% min), THF (Thermo Fisher Scientific, 99.7%), 2-mercaptopropionic acid (Sigma-Aldrich, 95.0% min), trifluoracetic acid (Sigma-Aldrich, 99.0% min), acetonitrile (Fisher Scientific, 99.9% min), 7-hydroxycoumarin (Comni-Blocks, 98.0%, min), 11-bromo-1-undecanol (Combi-Blocks, 98.0% min), anhydrous potassium carbonate (Chem-Supply, 99.0% min), potassium iodine (Chem-Supply, 99.0%), acetone (Themo Fisher Scientific, 99.0% min), oxalyl chloride (Sigma-Aldrich, 98.0% min), dimethylformamide (Thermo Fisher Scientific, 99.8% min), pyridine (Sigma-Aldrich, 99.0% min).

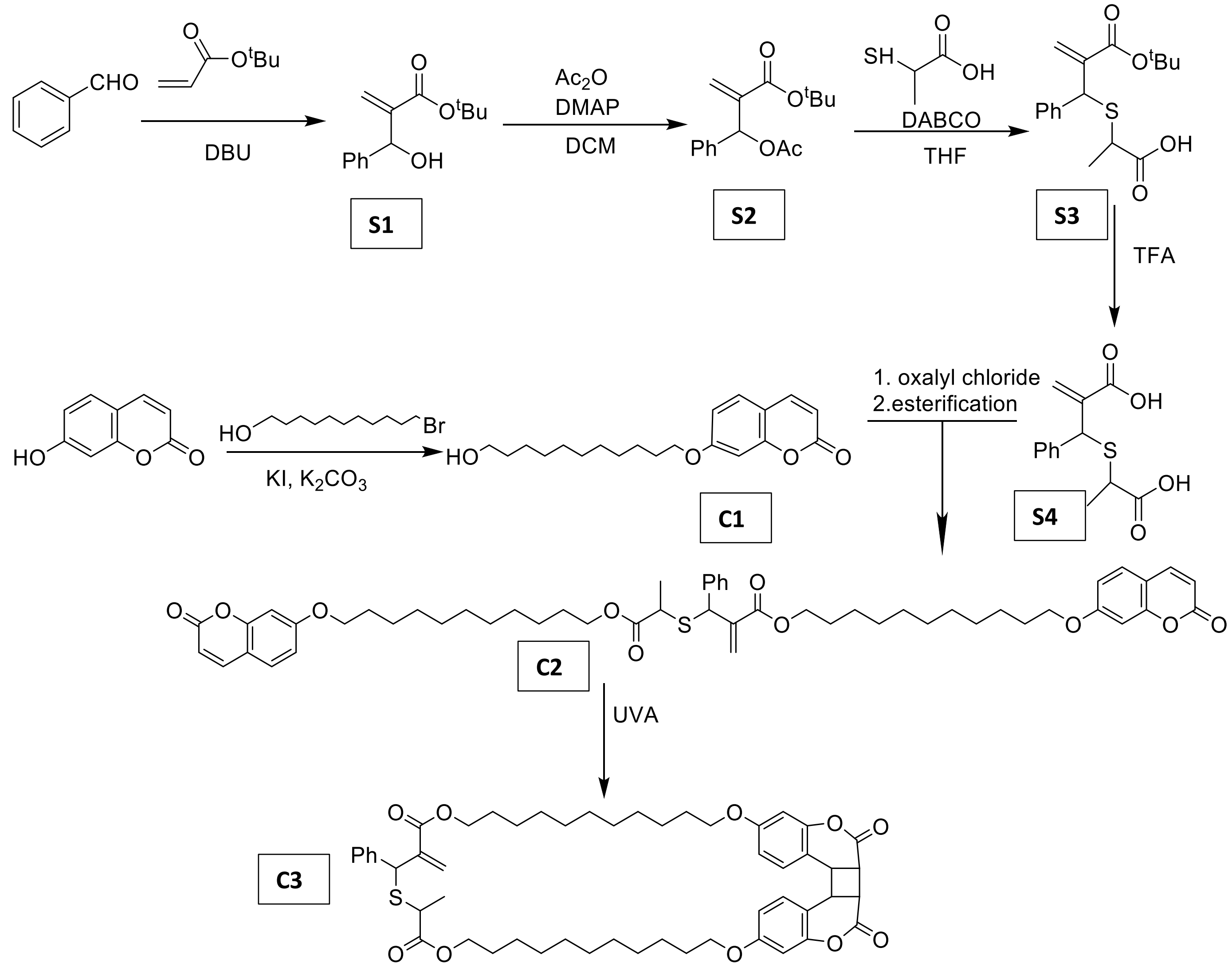

Scheme S 1. Synthetic route of macro cyclic monomer C3

3.2. Synthesis

Synthesis of **S1-S4** was conducted following the procedures described elsewhere [3] with a slight modification.

3.2.1. S1

Benzaldehyde (5.3 g, 50.0 mmol, 1.0 eq), t-butyl acylate (6.4 g, 50.0 mmol, 1.0 eq) and 1,8-Diazabicyclo [5.4.0] undec-7-ene (4.6.3 g, 30.0 mmol, 0.6 eq) were added to a round-bottom flask. The reaction mixture was stirred at room temperature for 100h. The mixture was then diluted with ethyl acetate (20 mL) and washed with HCl 3M (30 mL x 3), brine (40 ml) and dried by Na_2SO_4 drying agent. The solvent and t-butyl acrylate were removed from

the mixture under reduced pressure at 40 °C to give yellow liquid containing benzaldehyde and S1 (74.4 wt%). A small amount of stabilizer 4-methoxyphenol was added into product before storage.

Yield: 7.75 g mixture containing **S1** (5.764 g, 24.6 mmol, 49.2% yield) and benzaldehyde.

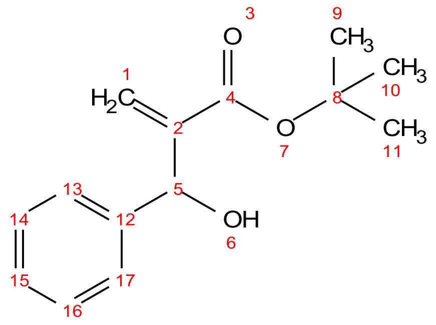

^{1}H NMR (600 MHz, CDCl3) δ 7.38 – 7.27 (m, 5H), 6.25 (s, 1H), 5.72 (s, 1H), 5.50 (s, 1H), 1.40 (s, 9H).

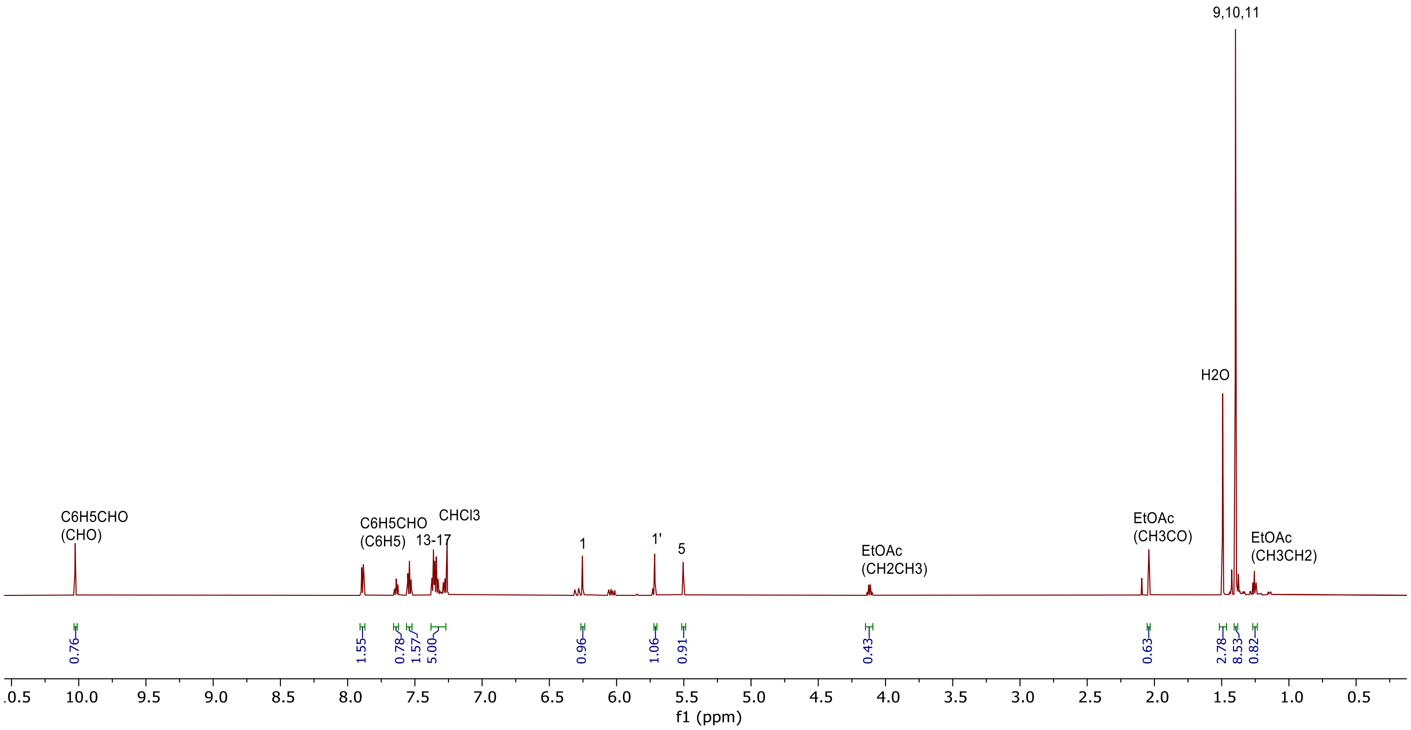

*Figure S 9. ^{1}H NMR spectrum of **S1** in $CDCl_3$*

3.2.2. S2

Acetic anhydride (3.0 g, 29.6 mmol, 1.2 eq), N, N-dimethylaminoprydine (0.6 g, 4.9 mmol, 0.2 eq) were added into an ice-cooled mixture of S1 and benzaldehyde obtained from the previous reaction (S1: 24.63 mmol, 1 eq) in dichloromethane (10 mL). The ice bath was then removed, and the reaction mixture was left stirring at room temperature for 16h. The reaction mixture was concentrated under reduced pressure at 40 °C and purified by flash column (solvent 20% ethyl acetate and 80% cyclohexane) to give yellow liquid containing S2 (84.2 wt%) and benzaldehyde. A small amount of stabilizer 4-methoxyphenol was added into product before storage.

Yield: 6.03 g mixture containing S2 (5.074 g, 18.28 mmol, 74.6% yield) and benzaldehyde.

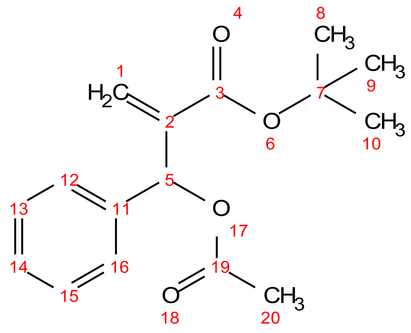

^{1}H NMR (600 MHz, $CDCl_3$) δ 7.37 – 7.28 (m, 5H), 6.63 (s, 1H), 6.32 (t, *J* = 1.2 Hz, 1H), 5.72 (t, J = 1.2 Hz, 1H), 2.10 (s, 3H), 1.37 (s, 9H).

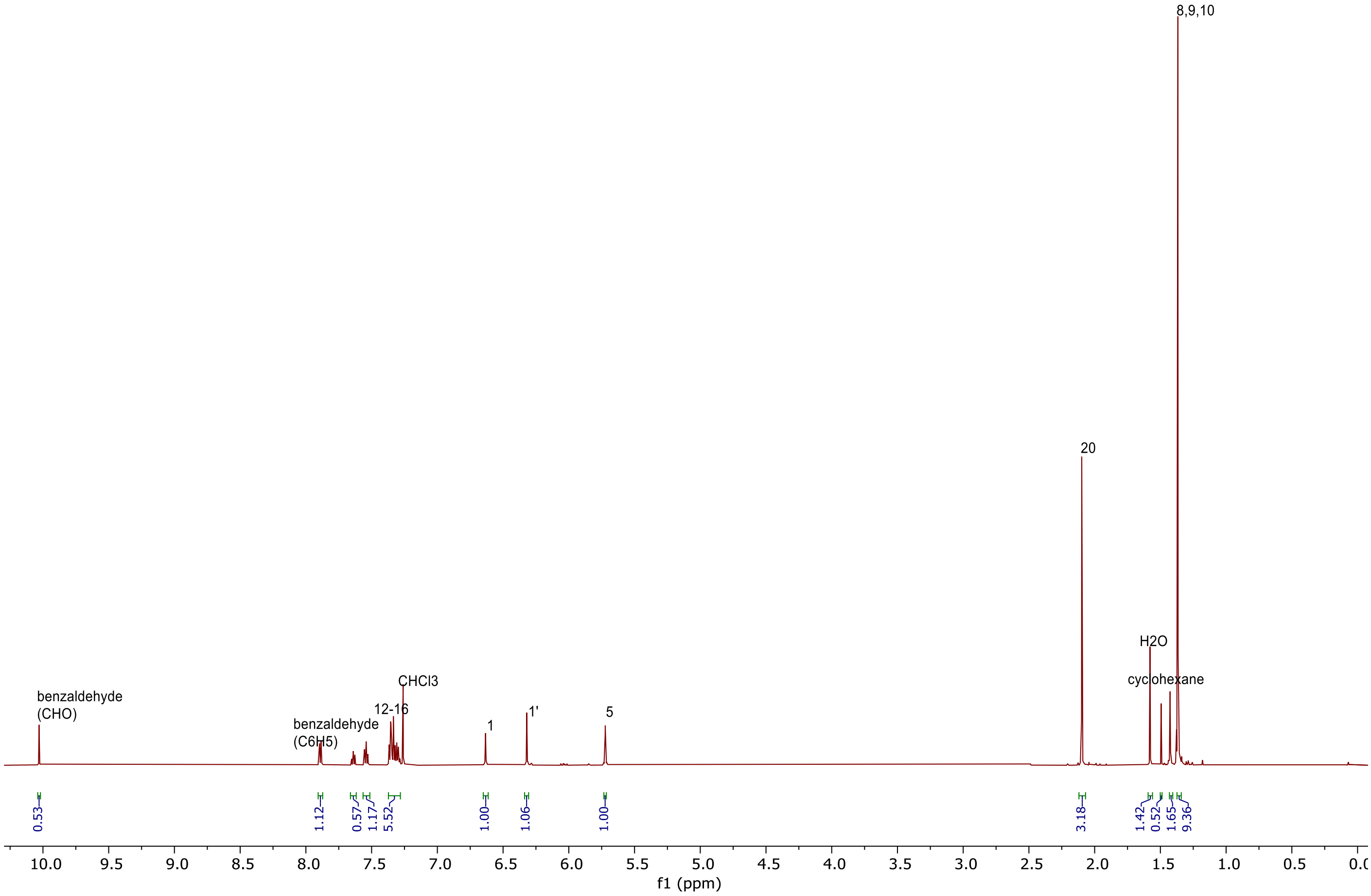

Figure S 10. ^{1}H NMR of **S2** in $CDCl_3$.

3.2.3. S3

Mixture A was prepared by adding 1,4-Diazabicyclo [2.2.2] octane (DABCO) (1.2 g, 11.0 mmol, 1.2 eq) to a well-stirred solution of S2 (9.1 mmol, 1.0 eq) and benzaldehyde obtained from the previous reaction in 5 mL of THF solvent. The mixture was stirred at room temperature for 1h. To solution of 2-mercaptopropionic acid (1.0 g, 9.1 mmol, 1.0 eq), and DABCO (1.0 g, 9.1 mmol, 1.0 eq) in 3.5 mL THF solvent, mixture A was added at room temperature. The reaction mixture was left stirring for 1.5h. THF was then removed by rotary-evaporator at 40 °C. The product mixture was purified by flash column chromatography to give colourless oil S3. A small amount of stabilizer 4-methoxyphenol was added to the product before storage.

Isolated yield: 1.2 g, 3.7 mmol, 40.9%.

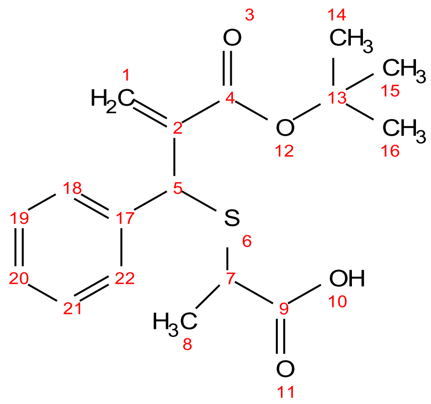

^{1}**H NMR** (600 MHz, $CDCl_3$) δ 7.43 – 7.37 (m, 2H), 7.37 – 7.26 (m, 3H), 6.32 (s, 1H), 5.83 (s, 1H), 5.24 (s, 1H), 3.15 (q, *J* = 7.2 Hz, 1H), 1.40 (s, 9H), 1.37 (d, *J* = 7.2 Hz, 3H).

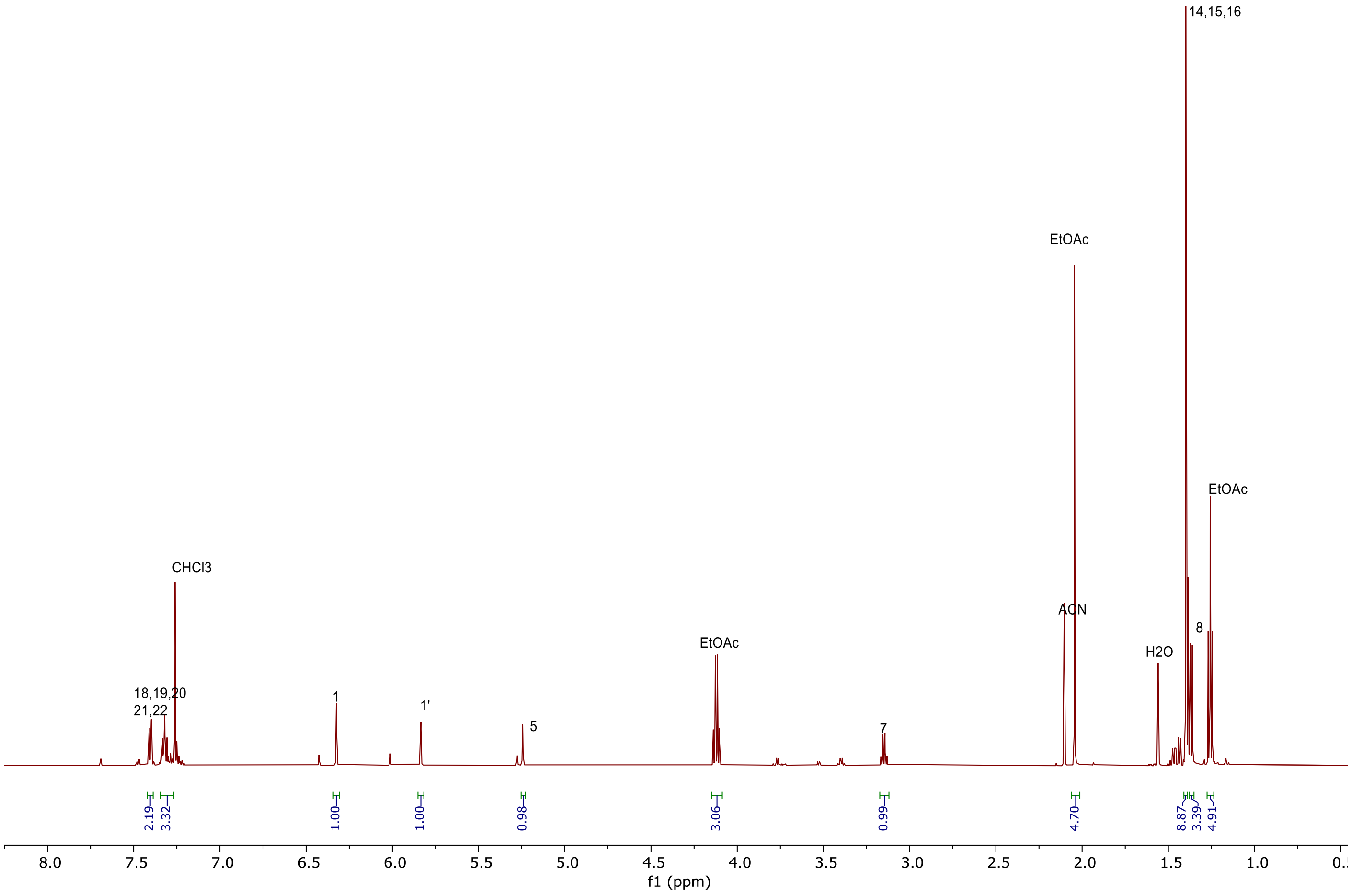

Figure S 11. ^{1}H NMR of **S3** in $CDCl_3$.

3.2.4. S4

S3 (1.2 g, 3.7 mmol, 1.0 eq) and 26mL of dichloromethane were added in a round-bottom flask. To the flask, trifluoracetic acid (TFA) (2.9 mL, 37.4 mmol, 10.0 eq) was added with stirring. The reaction mixture was left stirring for 4.5 h at room temperature. After that, 60 mL of acetonitrile was added to the mixture. All volatile components were removed by concentrating the mixture under reduced pressure to give a light brown oil S4. A small amount of stabilizer 4-methoxyphenol was added into product before storage.

Isolated yield: 3.74 mmol, 996 mg, 100%.

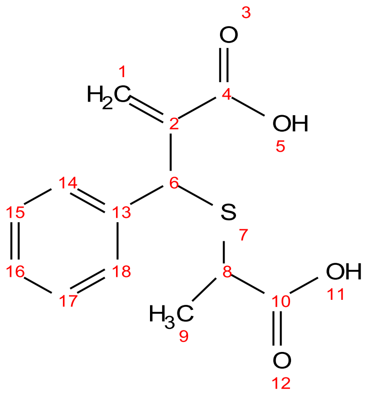

LC-ESI-HRMS negetive mode: $C_{13}H_{13}O_4S^-$ m/z=265.0540; found: 265.0538, $\Delta m/z$ =-0.75ppm.

^{1}H NMR (600 MHz, $CDCl_3$) δ 7.48 (d, J = 7.7 Hz, 2H), 7.42 – 7.35 (m, 3H), 6.46 (s, 1H), 5.70 (s, 1H), 5.40 (s, 1H), 3.24 (q, J = 7.2 Hz, 1H), 1.37 (d, J = 7.3 Hz, 3H).

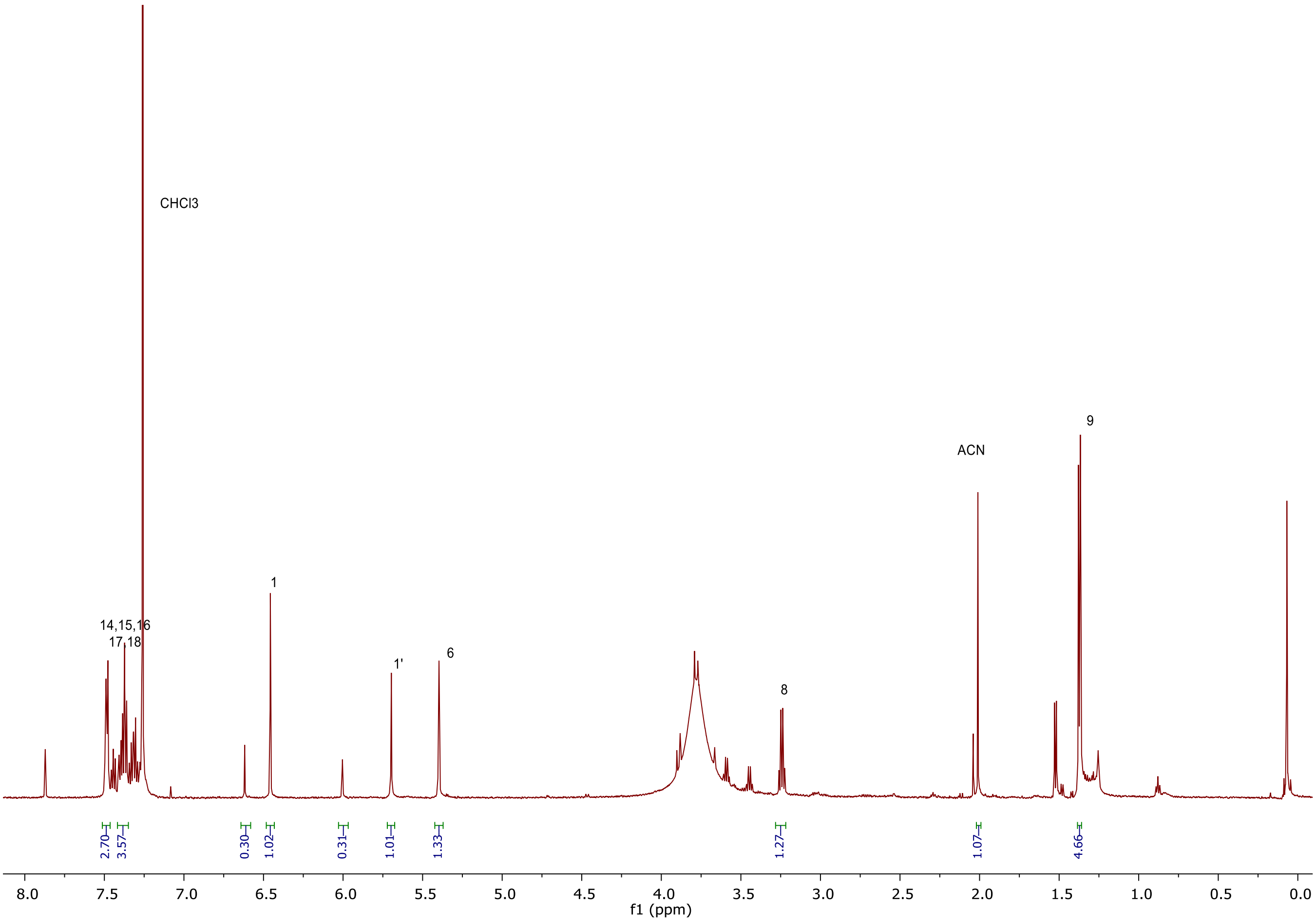

Figure S 12. ^{1}H NMR of **S4** in $CDCl_3$.

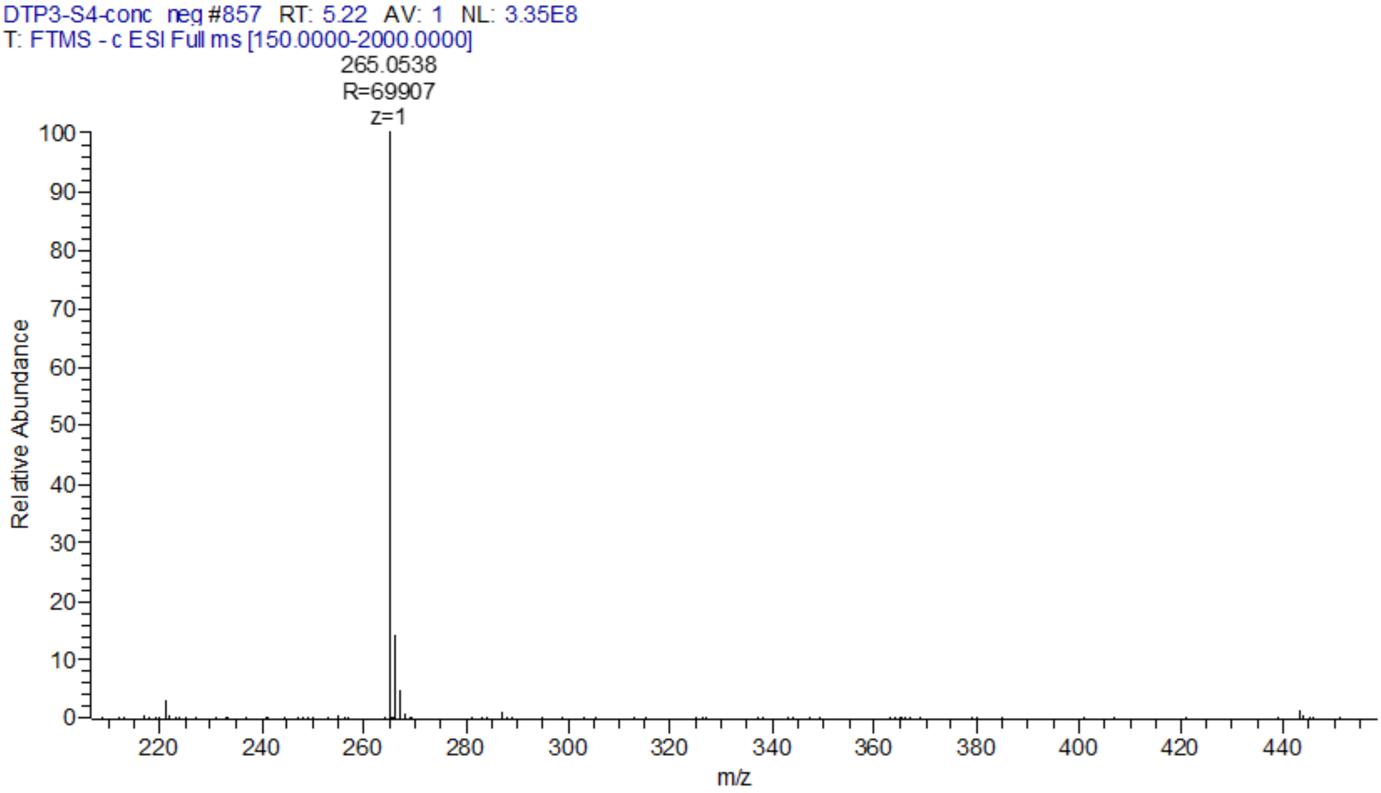

*Figure S 13. HRMS spectrum of **S4** from LC-HRMS.*

3.2.5. C1

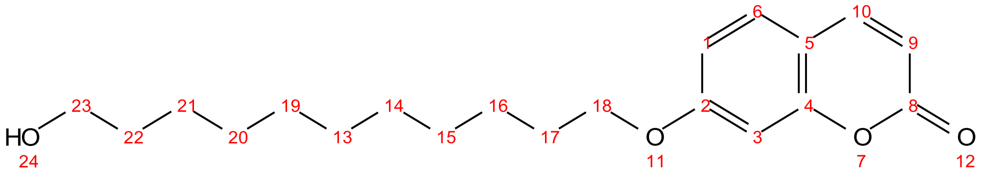

The synthesis procedure was adopted from literature.[4] 7-hydroxycoumarin (1.9 g, 12.0 mmol, 1.0 eq) ,11-Bromo-1-undecanol (3.0 g, 12.0 mmol, 1.0 eq), K_2CO_3 (5.5g. 40.0 mmol, 3.3 eq), 30 mg KI and 75 mL freshly distilled acetone were added to a round-bottom flask. The reaction mixture was refluxed at 61 °C for 45 h. After reaction, the solid was filtered out and the solvent was removed under reduced pressure to afford a yellow solid. The solid was dissolved in ethyl acetate (200 mL) and then washed with HCl 3N (200 mL x3), brine (200 mL), and dried over Na_2SO_4. Ethyl acetate was removed under reduced pressure to afford a yellow solid C1.

Isolated yield: 3.85 g, 11.58 mmol, 96.5%.

LC-ESI-HRMS: $C_{20}H_{28}O_4H^+$ m/z= 333.2060; found: 333.2055, $\Delta m/z$ =1.5ppm.

^{1}H NMR (600 MHz, Acetone-D6) δ 7.89 (d, J = 9.5 Hz, 1H), 7.57 (d, J = 8.6 Hz, 1H), 6.92 (dd, J = 8.6, 2.4 Hz, 1H), 6.88 (d, J = 2.4 Hz, 1H), 6.20 (d, J = 9.5 Hz, 1H), 4.13 (t, J = 6.5 Hz, 2H), 3.55 – 3.47 (m, 2H), 1.86 – 1.78 (m, 2H), 1.54 – 1.25 (m, 16H).

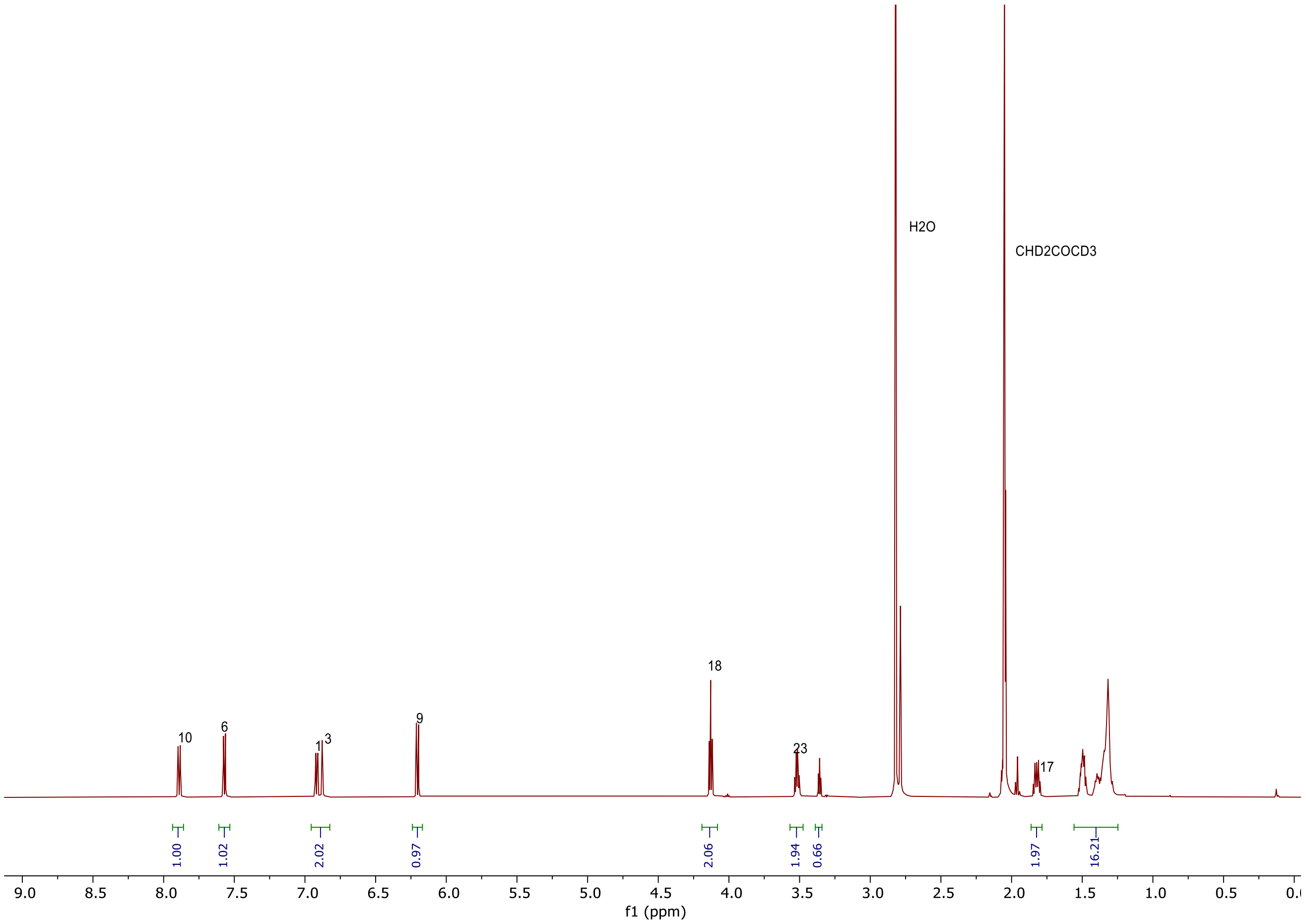

Figure S 14. ^{1}H NMR of **C1** in acetone-D_6

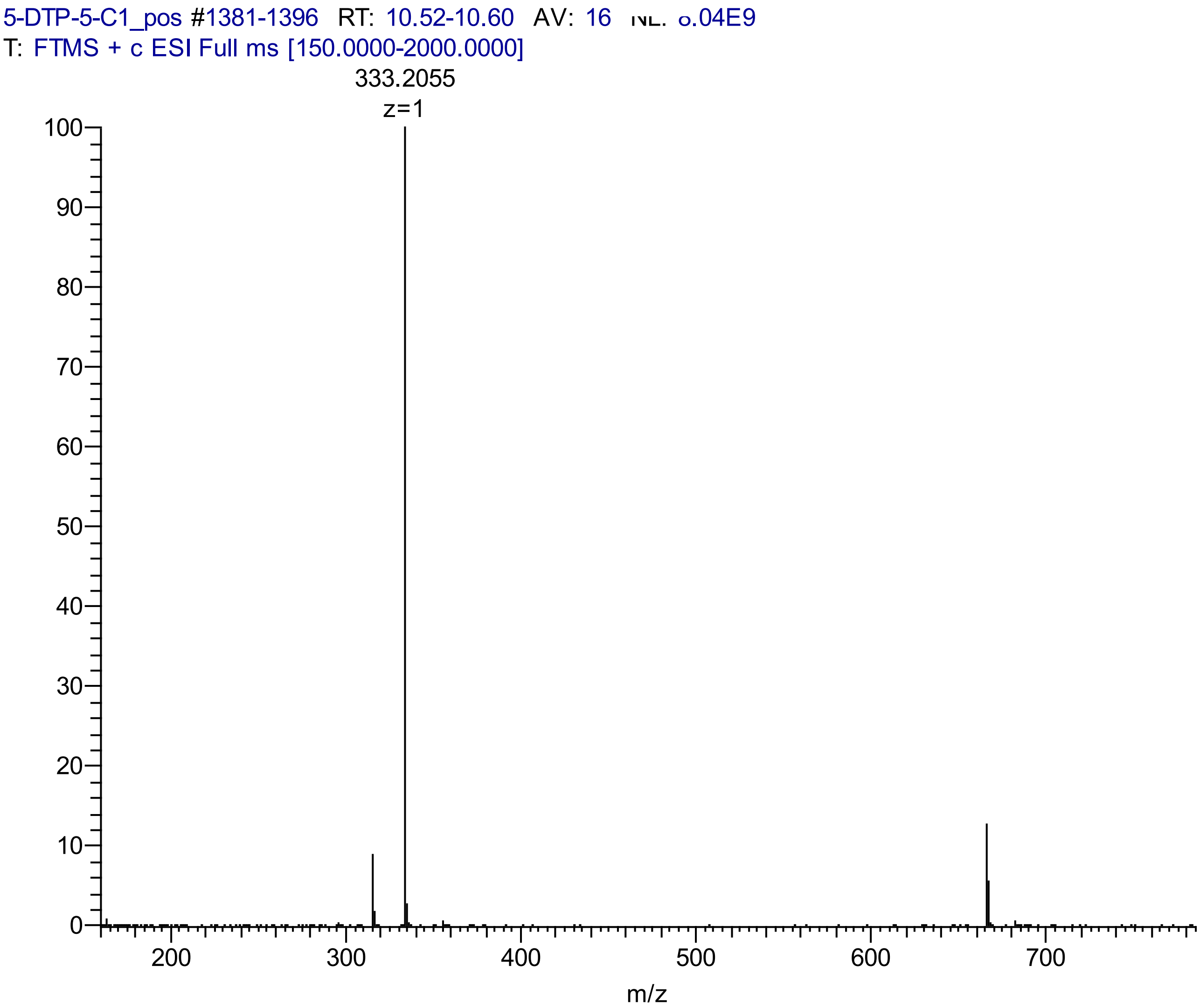

*Figure S 15. HRMS spectrum of **C1** from LC-HRMS.*

3.2.6. C2

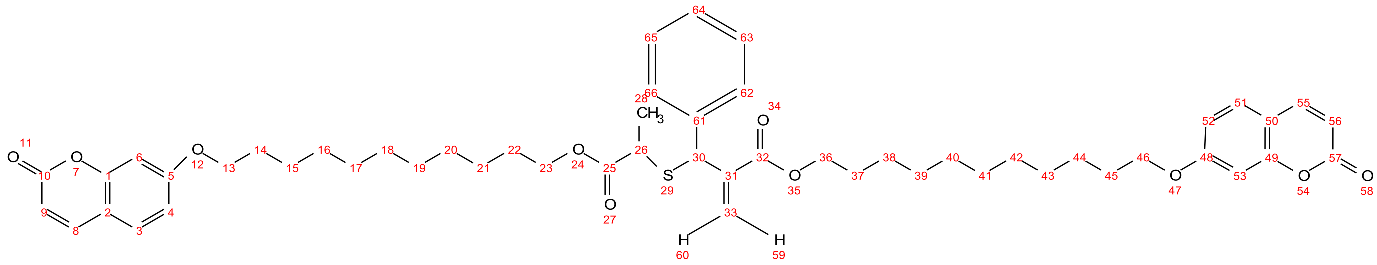

S4 (266.3 mg, 1.0 mmol, 1.0 eq), dry dichloromethane (5 mL), and oxalylchloride (0.3 mL, 4.0 mmol, 4.0 eq) were charged to an ice-cooled flask with stirring. 1 drop of dimethylformamide was then added to catalyse the reaction. The flask was stirred under Argon for 3h. After completion, the excess oxalyl chloride and dichloromethane solvent under reduced pressure. Subsequently, the flask was kept under Argon, and C1 (1.3 g, 4.0 mmol, 4.0 eq), pyridine (0.4 mL, 5.0 mmol, 5.0 eq) and dry acetonitrile (50 mL) were added with stirring. The reaction mixture was left stirring under Argon for 4h. After that, the reaction mixture was concentrated under reduced pressure and the residue was purified by a flash column chromatography and then by a reverse phase HPLC to give a yellow oil C2.

Isolated yield: 167.15 mg, 0.19 mmol, 19%.

^{1}**H NMR** (600 MHz, Acetone):

Isomer 1: δ 7.88 (d, *J* = 9.5 Hz, 2H), 7.56 (d, *J* = 8.6 Hz, 2H), 7.44 – 7.39 (m, 2H), 7.36 – 7.30 (m, 3H), 6.91 (ddd, *J* = 8.6, 2.4, 0.7 Hz, 2H), 6.86 (d, *J* = 2.4 Hz, 2H), 6.39 (s, 1H), 6.20 (d, *J* = 9.5 Hz, 2H), 5.98 (s, 1H), 5.36 (s, 1H), 4.15 – 4.01 (m, 8H), 3.22 (q, *J* = 7.2 Hz, 1H), 1.86 – 1.18 (m, 39H).
Isomer 2: δ 7.88 (d, *J* = 9.5 Hz, 2H), 7.56 (d, *J* = 8.6 Hz, 2H), 7.44 – 7.39 (m, 2H), 7.36 – 7.30 (m, 3H), 6.91 (ddd, *J* = 8.6, 2.4, 0.7 Hz, 2H), 6.86 (d, *J* = 2.4 Hz, 2H), 6.53 (s, 1H), 6.20 (d, *J* = 9.5 Hz, 2H), 6.18 (s, 1H), 5.38 (s, 1H), 4.15 – 4.01 (m, 8H), 3.41 (q, *J* = 7.2 Hz, 1H), 1.86 – 1.18 (m, 39H).

SEC-ESI-HRMS: $[C_{53}H_{66}O_{10}SNa]^+$ *m/z*= 917.4269; found: 917.4256, Δ*m/z*= 1.4ppm.

IM-MS: mass-selected m/z 917- ion $[C_{53}H_{66}O_{10}SNa]^+$. Differentiation of two isomers was observed after ions travel through 31 passes of cyclic ion mobility device. Drift time of isomer 1 and isomer 2 are 765.77 ms and 773.30 ms, respectively. The two isomers have identical mass and isotopic pattern.

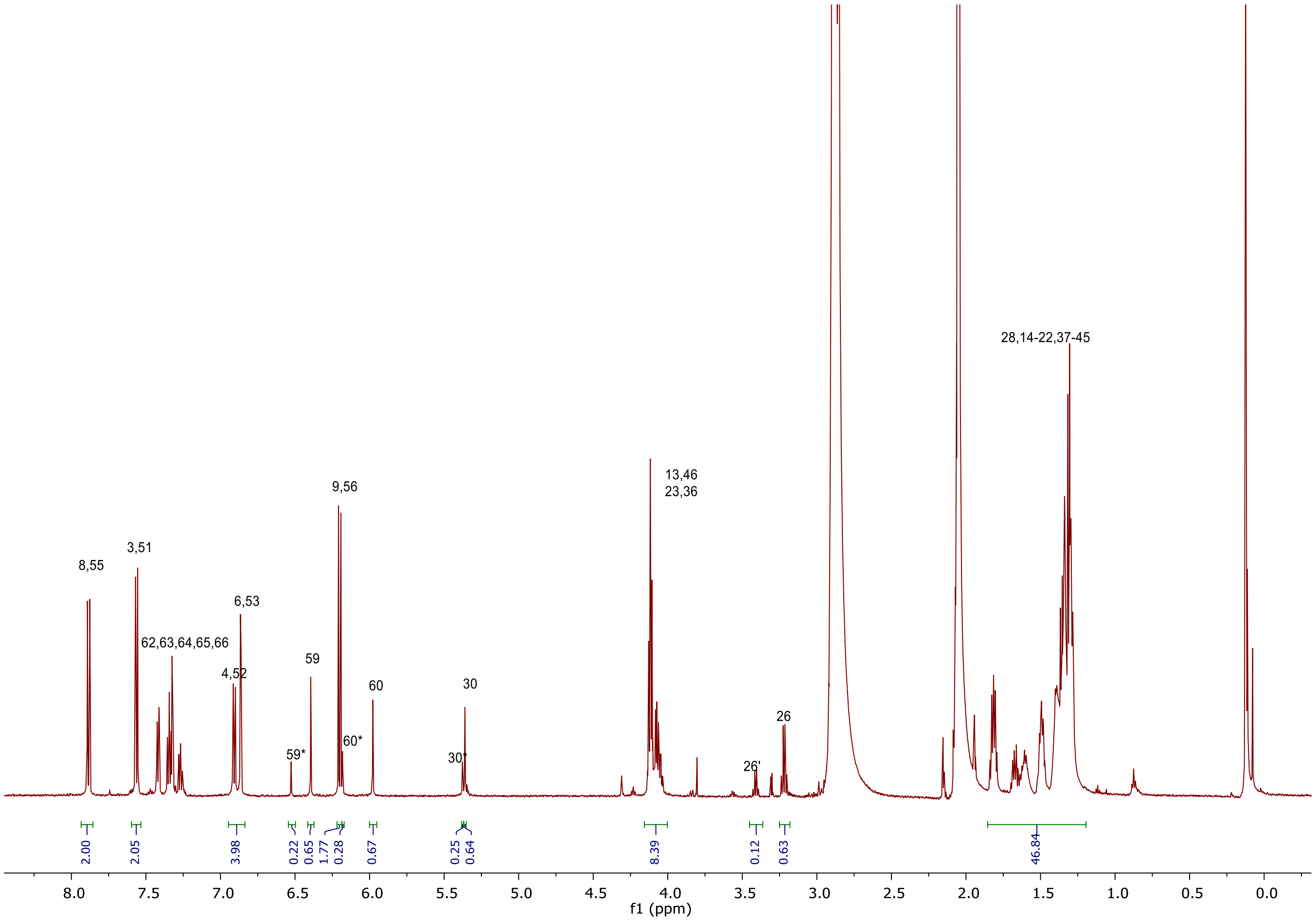

Figure S 16. ^{1}H NMR of **C2** in acetone-D_6.

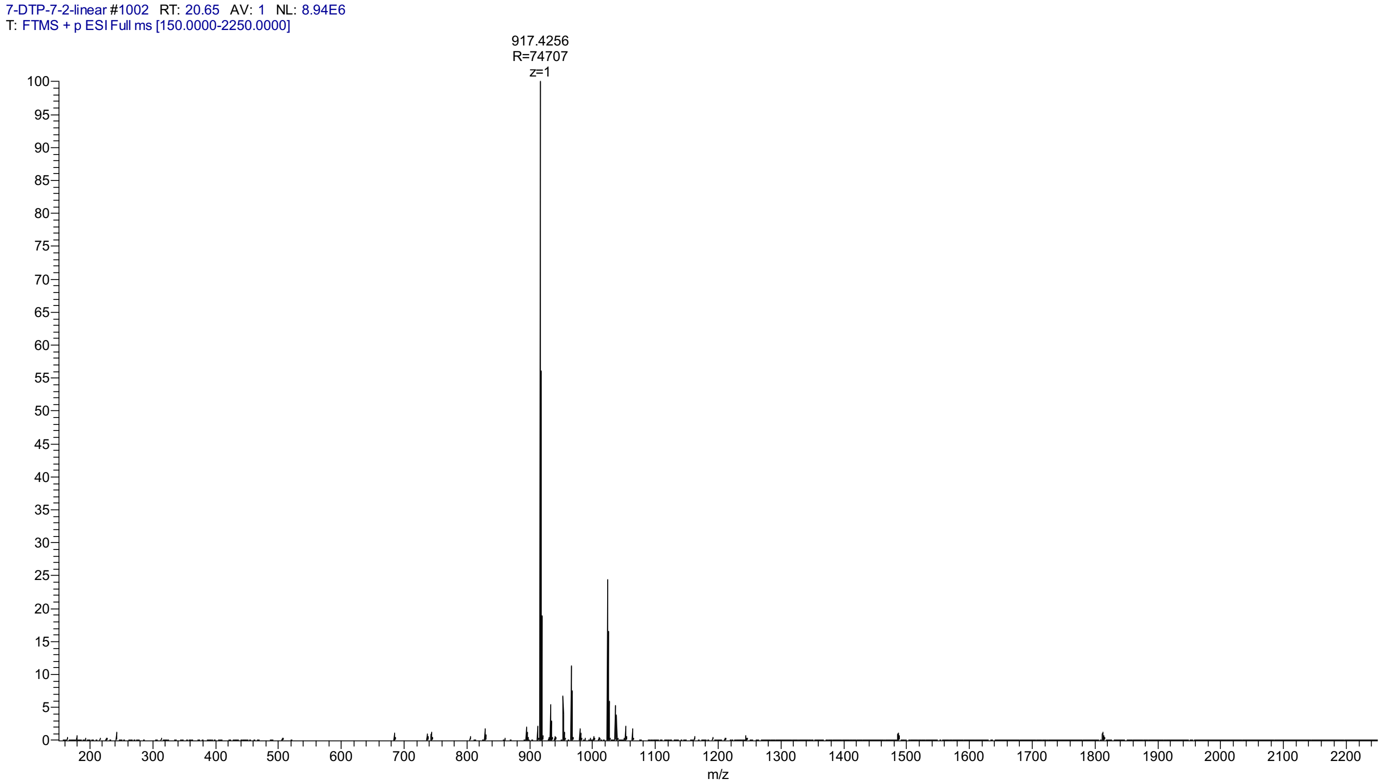

*Figure S 17. HRMS spectrum of **C2** from SEC-HRMS.*

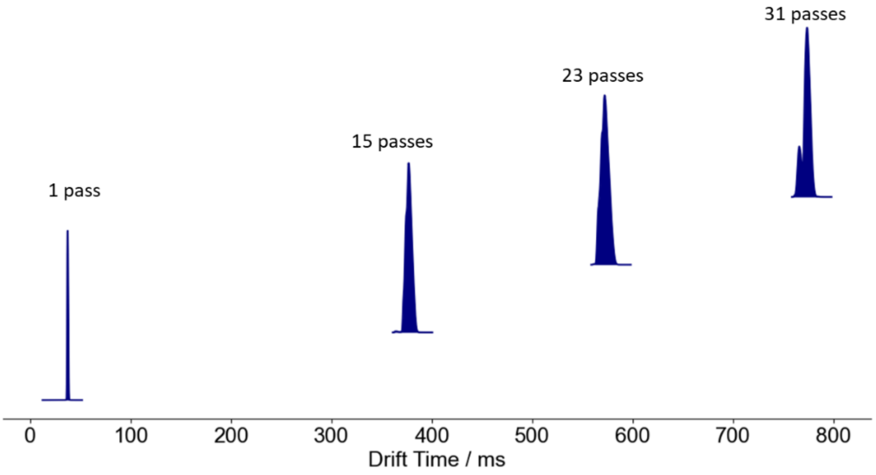

*Figure S 18. Drift time distribution of the linear monomer **C2** after the mass selected ion was separated after 1, 15, 23 and 31 passes around the cyclic IMS device.*

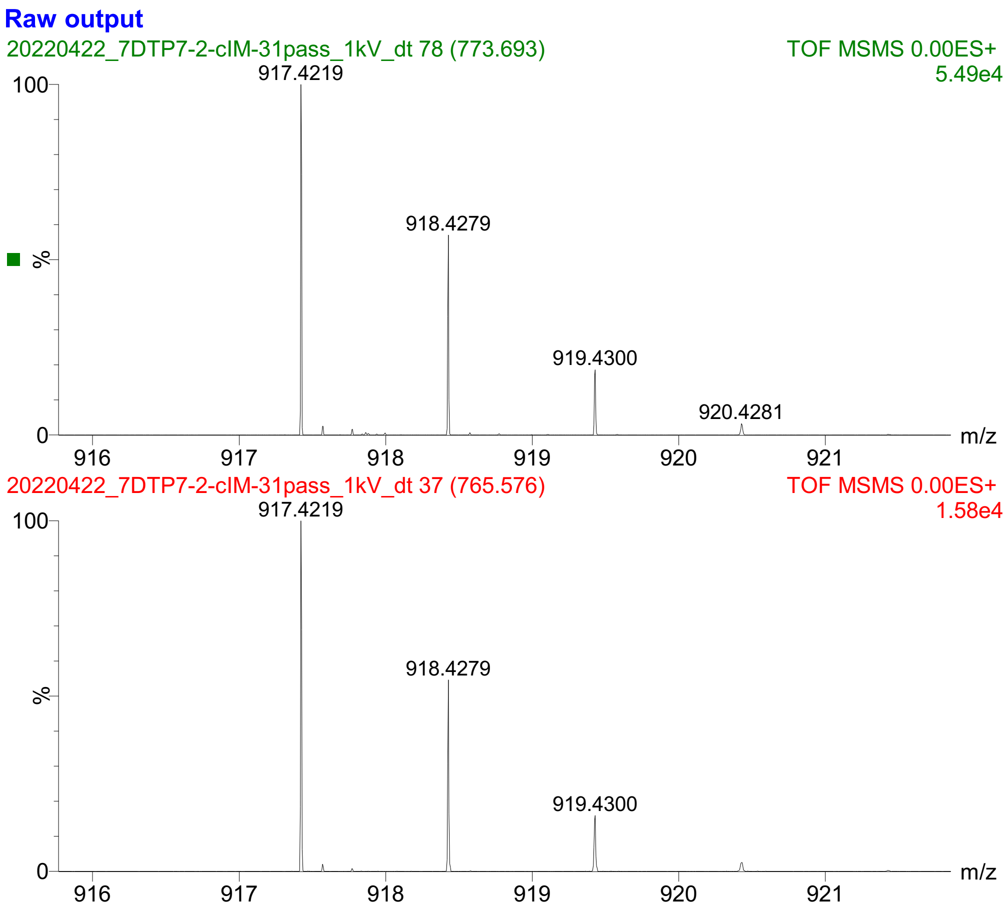

*Figure S 19. Mass spectra m/z 916-922 corresponding to drift time 773.69 ms (top) and 765.58 ms (bottom) when **C2** ions travel 31 passes in cyclic device.*

3.2.7. C3

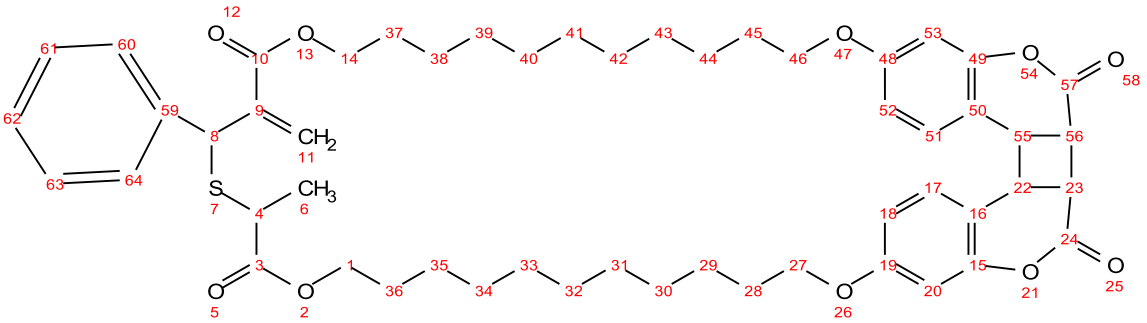

A solution of C2 in acetonitrile 2.0 mg. mL^{-1} (10 mL) was irradiated with UVA lamps. The dimerization of coumarin groups was monitored by UV-Vis and SEC trace with detector at λ = 267 nm, the isobestic point of coumarin and coumarin dimer. The photoreaction was stopped after 4 h of irradiation when all coumarin groups were consumed as indicated in UV-Vis spectrum and SEC chromatogram of reaction solution. The reaction mixture was concentrated by a rotary-evaporator and purified with HPLC-reverse phase column to give the colourless oil C3.

Isolated yield: 14.61 mg; 0.0166 mmol, 73.4%.

SEC-ESI-HRMS: $[C_{53}H_{66}O_{10}SNa]^+$ *m/z*= 917.4269; found: 917.4259, Δ*m/z*= 1.1ppm

^{1}H NMR (600 MHz, Acetone) two isomers refer to isomers from [2+2] cyclobutane addition whose ratio are (1:0.8).

Isomer 1: δ 7.44 (d, *J* = 1.3 Hz, 2H), 7.38 – 7.30 (m, 3H), 6.96 (d, *J* = 8.5 Hz, 2H), 6.67 (d, *J* = 1.0 Hz, 2H), 6.40 (s, 1H), 6.10 (d, *J* = 1.8 Hz, 2H), 5.98 (d, *J* = 5.0 Hz, 1H), 5.38 (s, 1H), 4.28 – 4.2 (m, 4H), 4.17 – 4.04 (m, 8H), 3.22 (q, *J* = 1.3 Hz, 1H), 1.76 – 1.19 (m, 39H).

Isomer 2 (*) δ 7.44 (d, *J* = 1.3 Hz, 2H), 7.38 – 7.30 (m, 3H), 6.84 – 6.76 (m, 2H), 6.55 – 6.50 (m, 2H), 6.40 (s, 1H), 6.36 (d, *J* = 1.2 Hz, 2H), 5.98 (d, *J* = 5.0 Hz, 1H), 5.38 (s, 1H), 4.24 – 4.21 (m, 2H), 4.15-4.12 (m, 2H), 3.97 – 3.90 (m, 8H), 3.22 (q, *J* = 1.3 Hz, 1H), 1.76 – 1.19 (m, 39H).

IM-MS: mass-selected m/z 917- nominal mass of ion $[C_{53}H_{66}O_{10}SNa]^+$. First differentiation of two components (two peaks) was observed after ions travel through 5 passes of cyclic ion mobility device, giving the drift time of 123.94 ms and 128.23 ms. The second peak was sliced out to pre-array storage then reinjected to cyclic device while other ions were disposed. After 20 passes of cyclic device, a differentiation of 3 components corresponding to 3 peaks of arrival time distribution of drift time was observed with drift time of 620.91 ms, 628.17 ms, 634.11 ms. In total, 4 isomers were observed by ion mobility- mass spectrometer evidenced by different drift times, and identical mass and isotopic patterns.

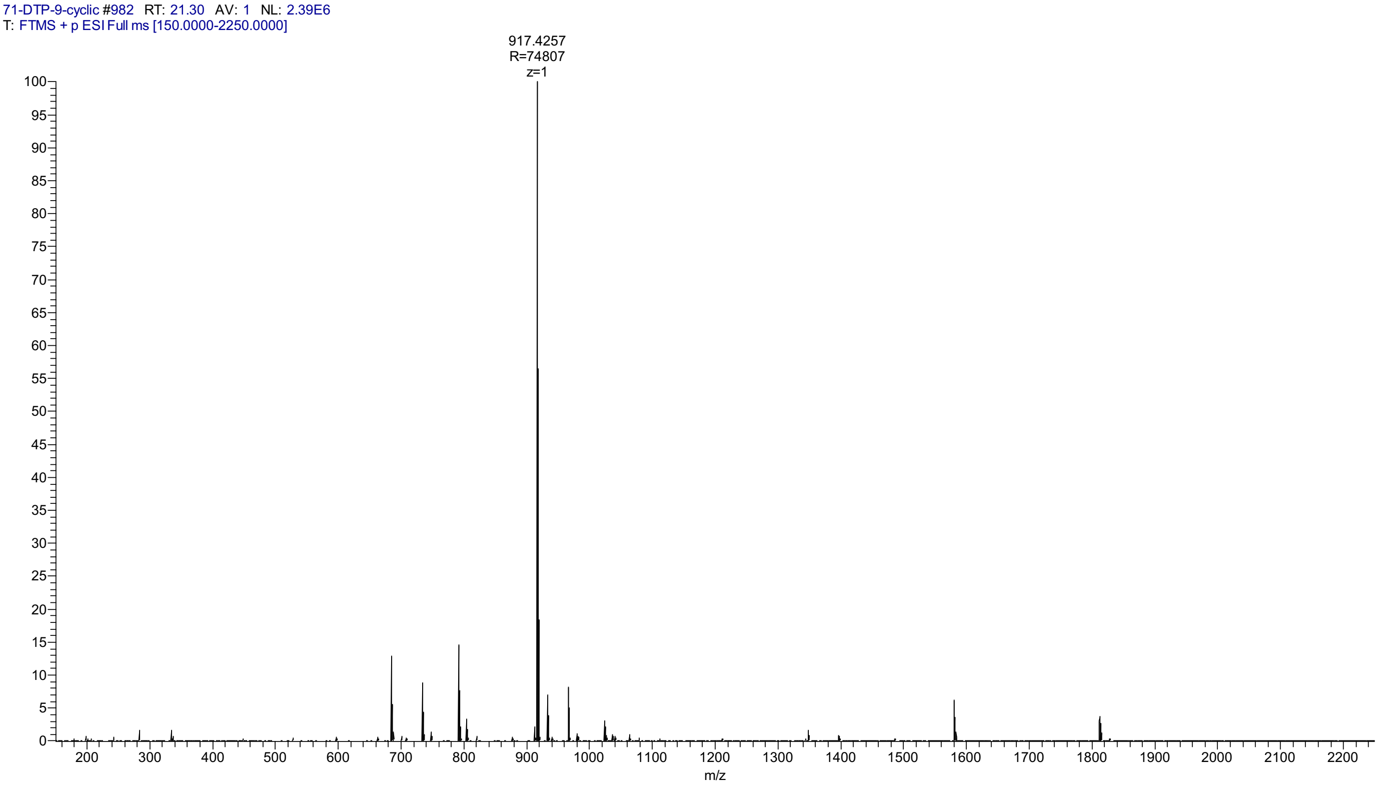

*Figure S 20. HRMS spectrum of **C3** from SEC-HRMS.*

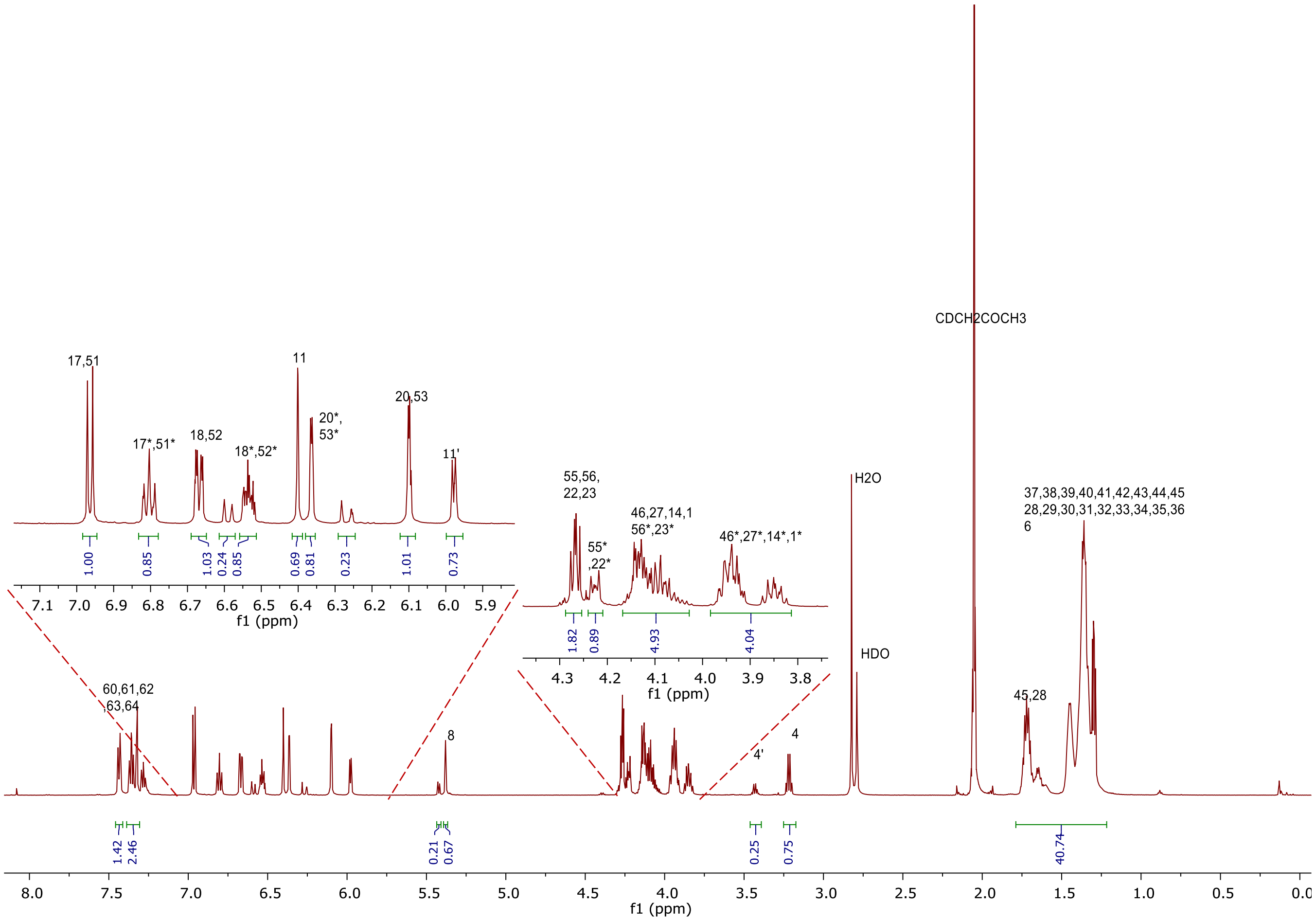

Figure S 21. ^{1}H NMR of **C3** in acetone-D$_6$

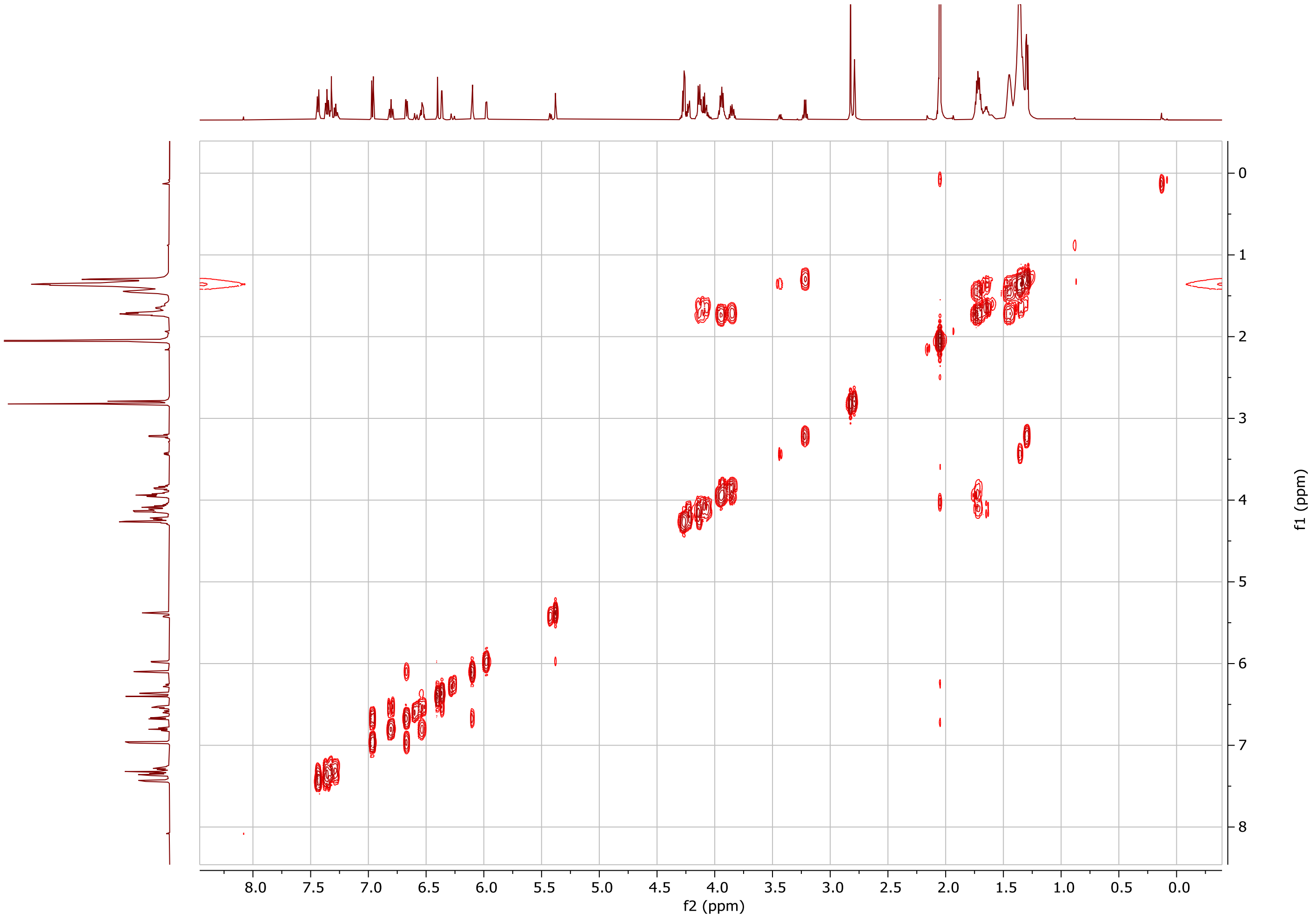

Figure S 22. COSY NMR of **C3** in acetone-D_6

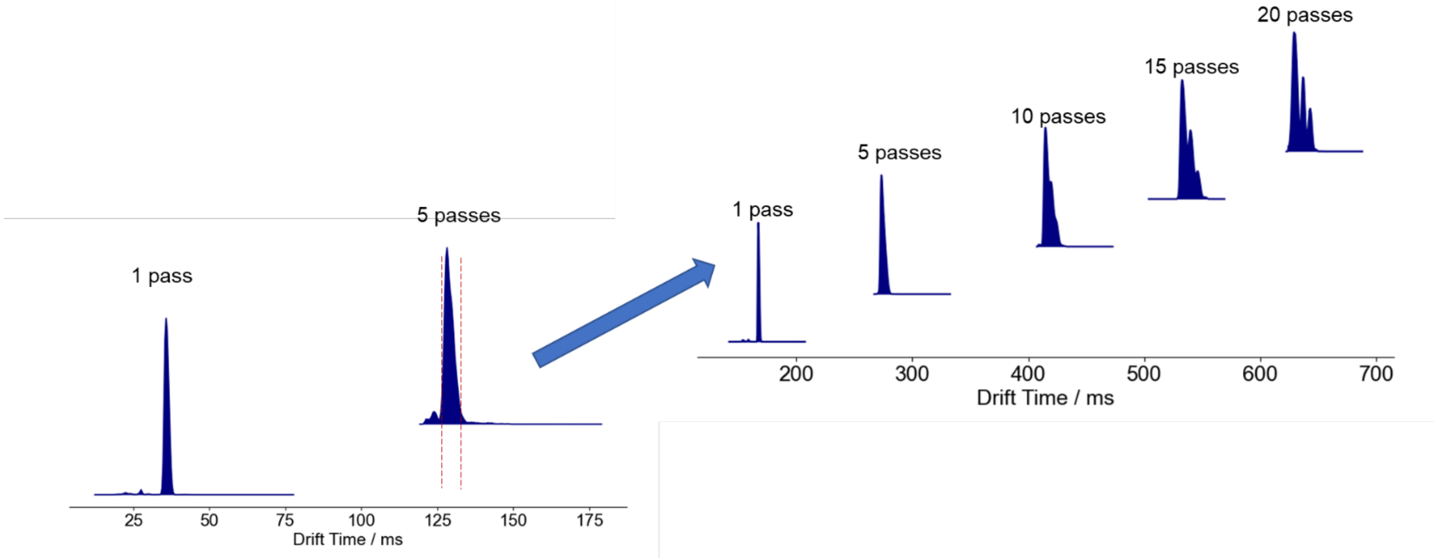

*Figure S 23. Drift time distribution of the cyclic monomer **C3** after the ions travel through cyclic device 1 pass, 5 passes and subsequently "sliced" ions travel through cyclic device 1 pass, 5 passes, 10 passes, 15 passes and 20 passes.*

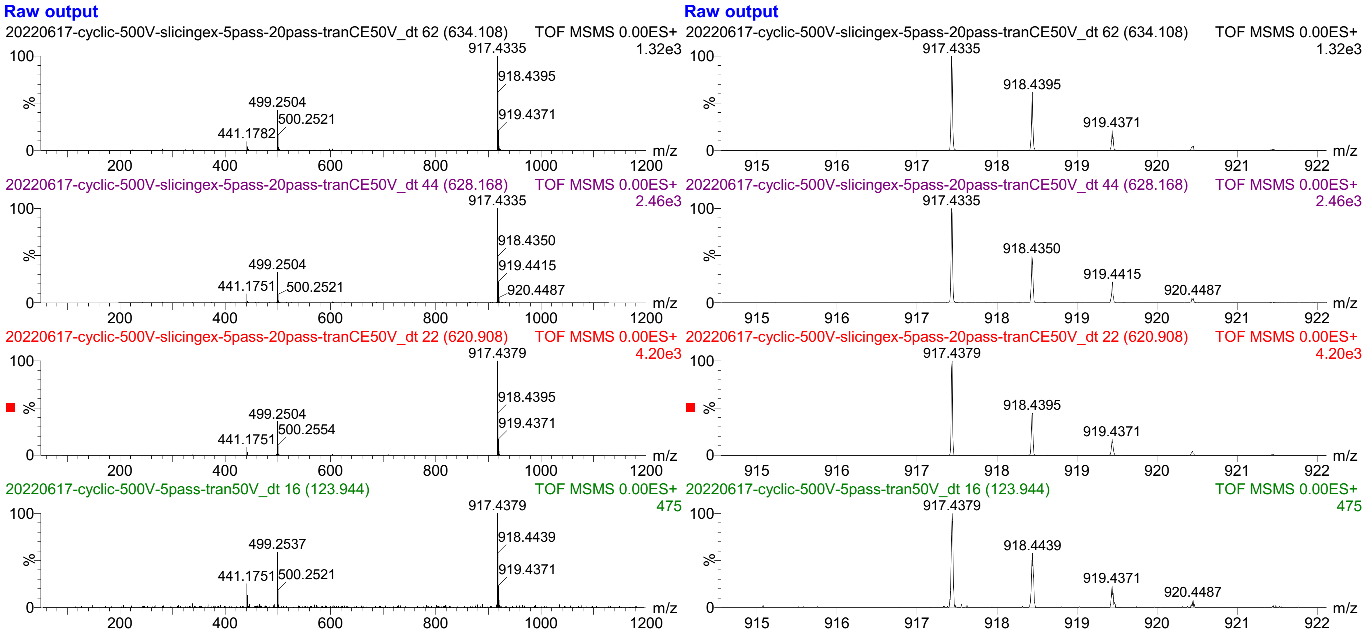

Figure S 24. Full mass spectra (left) and extracted mass spectra m/z 915-922 (right) corresponding to drift time (top to bottom order) 634.11 ms, 628.17 ms, 620.91 ms, and 123.94 ms with collision energy 50V applied at transfer region from cyclic device to TOF detector.

4. Polymer synthesis

4.1. Material

Methyl acrylate (Sigma-Aldrich, 99.0% min, after passing through a short plug of basic alumina), 2-(Dodecylthiocarbonothioylthio) propionic acid (synthesized according to literature[5]), Azobisisobutyronitrile (Sigman-Aldrich-Merck, recrystalized), Toluene (Thermo Fisher Scientific), Hexane (Thermo Fisher Scientific).

4.2. Synthesis

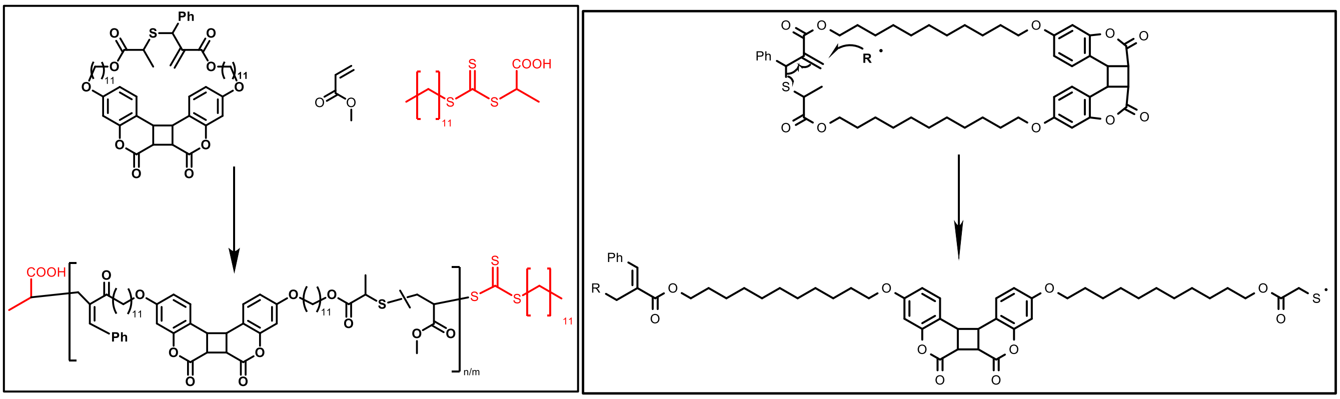

*Scheme S 2. Chemical reaction of copolymerisation of cyclic monomer **C3** and MA (left) and ring-opening mechanism of cyclic monomer **C3** (right).*

4.2.1. Synthesis of polymer

Synthesis of polymer P1 ([MA]/[CTA] = 200, [C3]/MA] = 2%)

Methyl acrylate monomer (54.4 µL, 600 µmol, 1000 eq), cyclic monomer C3 (10.73 mg, 12 µmol, 20 eq), AIBN (19.7 µL of 5mg/mL AIBN in toluene solution, 0.6 µmol, 1 eq), 2- (Dodecylthiocarbonothioylthio) propionic acid (105.2 µL of 10 mg/mL in toluene solution, 3 µmol, 5 eq) were added in a vial with a magnetic stirrer. The vial was then capped and deoxygenated by purging Argon gas through for 15 minutes. The vial was left stirring at 70°C. After 2h, the vial was uncapped to stop the polymerisation. The reaction mixture was precipitated in hexane. The polymer precipitate was collected by centrifugation and dried under reduced pressure to give a yellow solid **P1**, (Molar weight Mn 15.2 kg.mol^{-1}, dispersity Đ = 1.37).

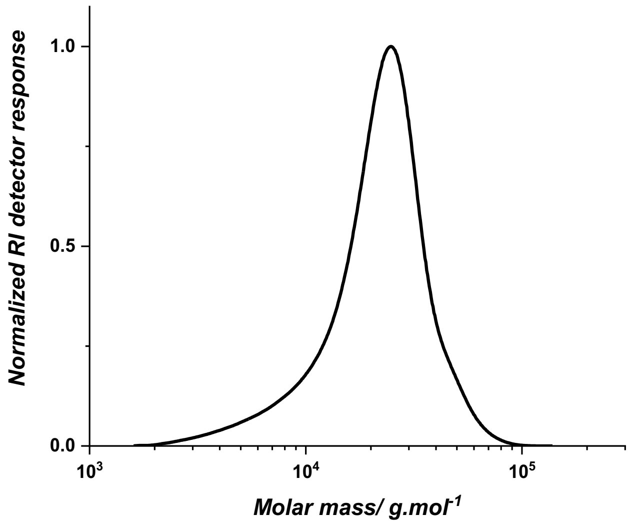

*Figure S 25. SEC chromatogram showing mass distribution of the copolymer **P1**.*

The control sample **P2** (homopolymer of MA) was prepared with the same procedure, but without the addition of cyclic monomer. The obtained polymer had a molecular weight of Mn = 11.6 kg mol^{-1}, dispersity *Đ* = 1.37.

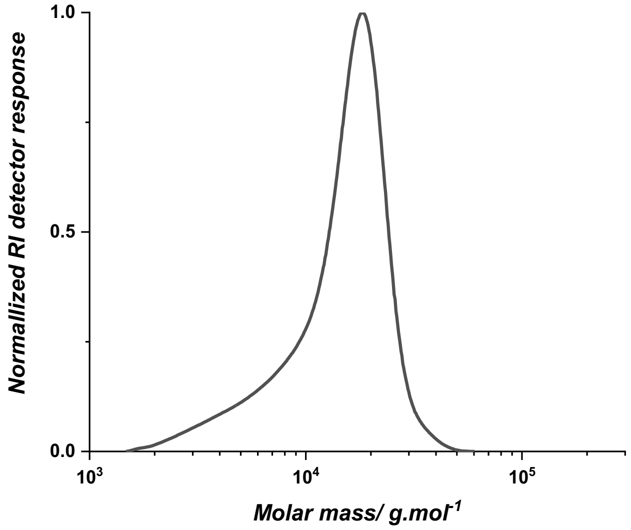

*Figure S 26. SEC chromatogram showing mass distribution of the homopolymer **P2.***

Synthesis of polymer P3 ([MA]/[CTA] = 400, [C3]/MA] = 1%)

Methyl acrylate monomer (106.4 µL, 1174 µmol, 2000 eq), cyclic monomer C3 (10.5 mg, 11.7 µmol, 20 eq), AIBN (19.3 µL of 5mg/mL AIBN in toluene solution, 0.6 µmol, 1 eq), 2- (Dodecylthiocarbonothioylthio) propionic acid (103 µL of 10 mg/mL in toluene solution, 3 µmol, 5 eq) and 122 µL toluene were added in a vial with a magnetic stirrer. The vial was then capped and deoxygenated by purging Argon gas through for 15 minutes. The vial was left stirring at 70°C. After 20h, the vial was uncapped to stop the polymerisation. The reaction mixture was precipitated in hexane. The polymer precipitate was collected by centrifugation and dried under reduced pressure to give a yellow solid **P3**, (MA conversion 86%, C3 conversion 88%, incorporation ration 0.8%, Molar weight Mn 26.8 kg.mol^{-1}, dispersity Đ = 1.33).

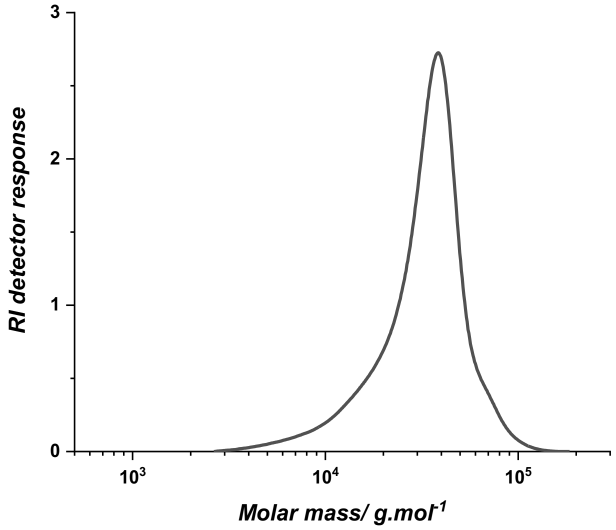

Figure S 27. SEC chromatogram showing mass distribution of the copolymer ***P3***

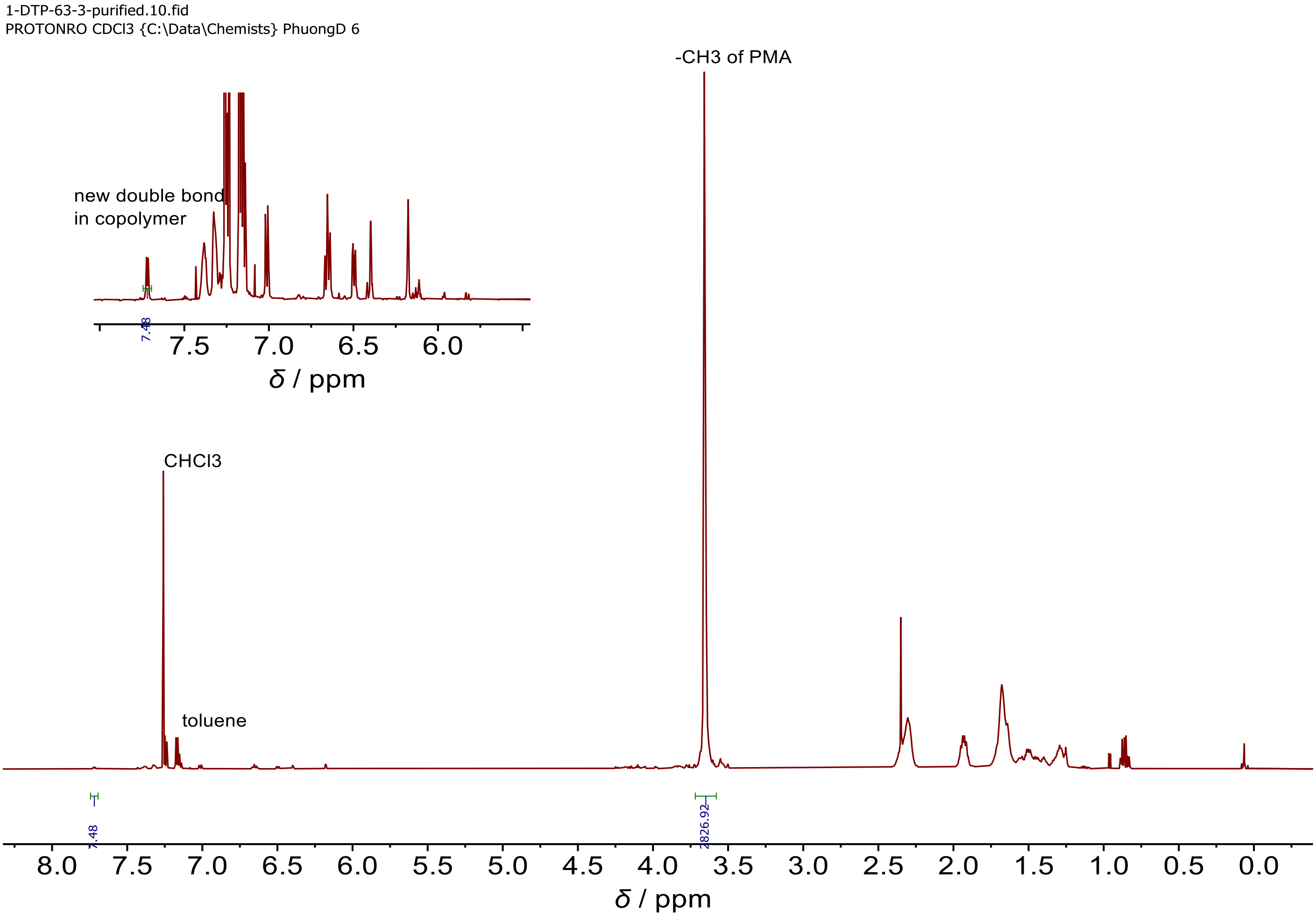

Figure S 28. ^{1}H NMR of **P3** in $CDCl_3$

4.2.2. Kinetic study of the copolymerisation

A reaction mixture of MA monomer (352.6 µL, 3891 µmol, 1000 eq), cyclic monomer **C3** (69.57 mg, 77.8 µmol, 20 eq), AIBN (127.8 µL of 5mg/mL AIBN in toluene solution, 3.9 µmol, 1 eq), 2- (Dodecylthiocarbonothioylthio) propionic acid (682.1 µL of 10 mg/mL in toluene solution, 19.5 µmol, 5 eq) was divided into equals parts of 90 µL and transferred into glass vials. These vials were then capped and deoxygenated by purging argon gas through for 7 minutes. After this, the vials were place in a heat plate at 70°C for different time periods corresponding to

different polymerisation times (10 min, 15 min, 20 min, 40 min, 60 min, 90 min, 120 min). To stop the polymerisation reaction, the vials were uncapped to expose reaction mixture to air. Conversion of monomers were identified by ^{1}H-NMR of crude reaction mixture after polymerisation. Molar weight and dispersity of product polymer were measured by THF-SEC. 13 µL of crude reaction mixture after polymerisation was diluted by 240 µL THF for SEC measurement.

Crude reaction mixtures with polymerisation time of 40 min, 60 min, 90 min, 120 min were then put under the vacuo for 5 days to eliminate MA residual and toluene solvent to afford yellow solid polymer **P4, P5, P6, P7**, respectively.

Monomer conversion analysis by NMR

Figure S24 below is ^{1}H NMR of crude reaction mixture after 90 min polymerisation. The peak p1 at δ = 3.66 ppm and the peak m at δ = 3.77 ppm correspond to methyl group of methyl acrylate polymer and methyl group of methyl acrylate monomer, respectively. The peak c'1 at δ = 5.98 ppm corresponds to one proton of the double bond in cyclic monomer C3 and the peak p2 δ = 7.72 ppm corresponds to proton of newly formed double bond in polymer of cyclic monomer (see Figure 2 for exact positions of protons mentioned).

conversion of MA monomer is calculated as follows: $MA\ conversion\ (\%) = \frac{p1}{p1+m} \times 100$

conversion of C3 monomer is calculated as follows: $C3\ conversion\ (\%) = \frac{p2}{p2+c'1} \times 100$

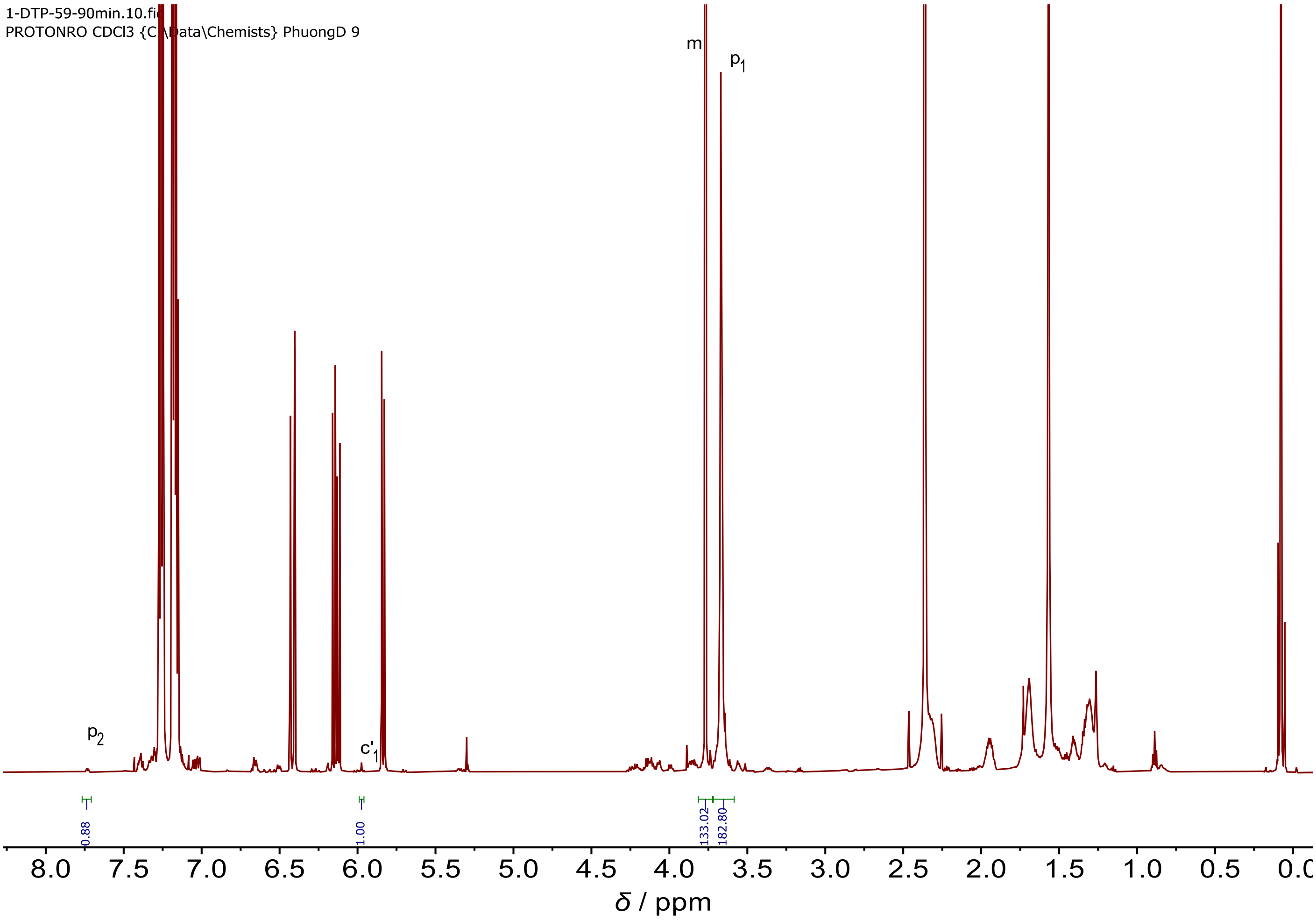

Figure S 29. ^{1}H NMR (CDCl3) of crude reaction mixture after 90 min polymerisation

1. Gruendling, T.; Guilhaus, M.; Barner-Kowollik, C., Fast and Accurate Determination of Absolute Individual Molecular Weight Distributions from Mixtures of Polymers via Size Exclusion Chromatography−Electrospray Ionization Mass Spectrometry. *Macromolecules* **2009,** *42* (17), 6366-6374.

2. Fulmer, G. R.; Miller, A. J.; Sherden, N. H.; Gottlieb, H. E.; Nudelman, A.; Stoltz, B. M.; Bercaw, J. E.; Goldberg, K. I., NMR chemical shifts of trace impurities: common laboratory solvents, organics, and gases in deuterated solvents relevant to the organometallic chemist. *Organometallics* **2010,** *29* (9), 2176-2179.
3. Huang, H.; Sun, B.; Huang, Y.; Niu, J., Radical Cascade-Triggered Controlled Ring-Opening Polymerization of Macrocyclic Monomers. *J Am Chem Soc* **2018,** *140* (33), 10402-10406.
4. Gindre, D.; Iliopoulos, K.; Krupka, O.; Evrard, M.; Champigny, E.; Salle, M., Coumarin-Containing Polymers for High Density Non-Linear Optical Data Storage. *Molecules* **2016,** *21* (2), 147.
5. Heiler, C.; Offenloch, J. T.; Blasco, E.; Barner-Kowollik, C., Photochemically Induced Folding of Single Chain Polymer Nanoparticles in Water. *ACS Macro Letters* **2017,** *6* (1), 56-61.
